# Role of CXCL5 in Regulating Chemotaxis of Innate and Adaptive Leukocytes in Infected Lungs Upon Pulmonary Influenza Infection

**DOI:** 10.3389/fimmu.2021.785457

**Published:** 2021-11-18

**Authors:** Lei Guo, Nan Li, Zening Yang, Heng Li, Huiwen Zheng, Jinxi Yang, Yanli Chen, Xin Zhao, Junjie Mei, Haijing Shi, G. Scott Worthen, Longding Liu

**Affiliations:** ^1^ Institute of Medical Biology, Chinese Academy of Medical Science and Peking Union Medical College, Kunming, China; ^2^ Yunnan Key Laboratory of Children's Major Disease Research, Yunnan Medical Research Center for Pediatric Diseases, Kunming Children’s Hospital, Kunming, China; ^3^ Division of Neonatology, Department of Pediatrics, Children’s Hospital of Philadelphia, Perelman School of Medicine, University of Pennsylvania, Philadelphia, PA, United States

**Keywords:** CXCL5, neutrophil, B lymphocyte, CXCL13, influenza, pulmonary infection

## Abstract

Respirovirus such as influenza virus infection induces pulmonary anti-viral immune response, orchestration of innate and adaptive immunity restrain viral infection, otherwise causes severe diseases such as pneumonia. Chemokines regulate leukocyte recruitment to the inflammation site. One chemokine CXCL5, plays a scavenging role to regulate pulmonary host defense against bacterial infection, but its role in pulmonary influenza virus infection is underdetermined. Here, using an influenza (H1N1) infected CXCL5^-/-^ mouse model, we found that CXCL5 not only responds to neutrophil infiltration into infected lungs at the innate immunity stage, but also affects B lymphocyte accumulation in the lungs by regulating the expression of the B cell chemokine CXCL13. Inhibition of CXCL5-CXCR2 axis markedly induces CXCL13 expression in CD64^+^CD44^hi^CD274^hi^ macrophages/monocytes in infected lungs, and *in vitro* administration of CXCL5 to CD64^+^ alveolar macrophages suppresses CXCL13 expression *via* the CXCL5-CXCR2 axis upon influenza challenge. CXCL5 deficiency leads to increased B lymphocyte accumulation in infected lungs, contributing to an enhanced B cell immune response and facilitating induced bronchus-associated lymphoid tissue formation in the infected lungs during the late infection and recovery stages. These data highlight multiple regulatory roles of CXCL5 in leukocyte chemotaxis during pulmonary influenza infection.

## Introduction

Respirovirus infection is one of the main causes of respiratory diseases worldwide. Every year, the influenza epidemic causes illness in millions of patients, and one in ten patients dies. Influenza virus enter the host through the upper respiratory tract, and directly infect airway epithelial cells, alveolar epithelial cells, and immune cells. Virus infection triggers cellular immune pathways to express abundant inflammatory cytokines and chemokines, including TNF-α, IL-1α/β, IL-2, IL-4, IL-6, IFN-α/β, CXCL-8 family members, CXCL-9/10, and MIP-1/2, in the respiratory tract and alveolar spaces (1, 2). These cytokines, in turn, recruit innate immune cells such as macrophages, granulocytes, dendritic cells (DCs), and natural killer (NK) cells into the infected lungs to exert anti-viral innate immune responses. Following infection, antigen-presenting cells (APCs) travel to the lung-draining lymph nodes and activate adaptive immune T cells and B cells. Activated lymphocytes then migrate to the infected lungs to exert their antiviral cellular immune and humoral immune effects, and ultimately clear viral infection and provide long-term protection (3). Fine-tuned pulmonary antiviral innate and adaptive immunity is critical for elimination of viral infection and control of lung inflammation; otherwise severe conditions such as pneumonia can develop.

Glutamic acid+leucine+arginine (ELR+) CXC chemokines are neutrophil chemoattractants. Seven members of this class of chemokines (IL-8 family) have been identified to date in humans, including IL-8 (CXCL8), CXCL1, CXCL2, CXCL3, CXCL5, CXCL6, and CXCL7 (4). No human CXCL8 homologs have been identified in rodents. However, CXCL1, CXCL2, CXCL3, CXCL5, and CXCL7 are important neutrophil chemoattractants in mice (5). Receptors for the IL-8 family include CXCR1, CXCR2, and Duffy Antigen Receptor for Chemokines (DARC); CXCR2 and DARC bind almost all the IL-8 family members (5, 6). CXCR1 and CXCR2 are members of the G protein-coupled receptor family that contain 7 transmembrane domains, and their expression can be detected on most leukocytes (at high levels on polymorphonuclear leukocytes) and structural cells, such as epitehlial and endothelial cells. Signals triggered *via* CXCR2 activate G protein and subsequent kinases to induce distinct biological outcomes that are often correlated with leukocyte migration and inflammation (7, 8). In contrast, DARC, which is highly expressed on the surfaces of erythrocytes and endothelial cells, lacks the intracellular motif to enable G protein coupling and is unable to transmit chemokine-mediated intracellular signals. Thus, DARC is considered a chemokine sink or a chemokine reservoir (9).

CXCL5, implicated in a variety of inflammatory diseases and tumorigenesis (10–12), is produced by lung resident cells, such as lung epithelial cells, and platelets during lung inflammation (13). A previous study by Mei et al. uncovered a critical role of CXCL5 in the control of chemokine scavenging and innate immunity against severe bacterial pneumonia (14). CXCL5 enhances the concentrations of other neutrophil chemokines in blood, in part by competing for binding to scavenger molecules, such as red cell DARC and heparan sulfate proteoglycans. During severe inflammation, high CXCL1 and CXCL2 plasma concentrations relative to those at the site of inflammation impair an effective chemokine gradient and desensitize CXCR2. Thus, CXCL5 negatively regulates neutrophil influx to the lung during severe *Escherichia coli* (*E. coli*) pneumonia by influencing the concentrations of other chemokines in the blood (14, 15). In addition to its prominent role in regulating innate immune cell trafficking in pulmonary bacterial infection models, how is CXCL5 function in respirovirus infection, such as influenza virus infection, which requires an intricate network of innate and adaptive immune mechanism. In this study, we generated an influenza (H1N1) infected CXCL5-knockout (CXCL5^-/-^) mouse model to elucidate the potential role of CXCL5 in viral infection-induced inflammatory pulmonary disease.

## Materials and Methods

### Mouse Infection Model

CXCL5^-/-^ mice (14) and normal C57BL/6 WT mice were obtained from Dr. G. Scott Worthen at the Children’s Hospital of Philadelphia. The mice were bred and housed in a barrier facility (in individually ventilated cages) at the Central Animal Care Services of the Institute of Medical Biology, Chinese Academy of Medical Sciences (CAMS), under specific-pathogen-free conditions. Male mice aged 10-12 weeks were used in all experiments. The mouse-adapted A/Puerto Rico/8/1934 H1N1 virus and A/Hong Kong/1968 H3N2 virus were stored in our laboratory. The viruses were grown in the chorioallantoic fluid of 9 d-old embryonated chicken eggs. The titers were determined from the CCID_50_ in Madin-Darby canine kidney (MDCK) cells using the method of Reed and Muench. WT and CXCL5^-/-^ mice were anesthetized by intraperitoneal injection of pentobarbital [50 mg per body weight (kg)] and then inoculated intranasally with 1000 or 3000 CCID_50_ of influenza virus in 30 μl of PBS (PBS alone was used as a control). Weight loss was monitored, and survival curves were generated. All animal experiments were conducted under prior approval from the animal ethics committee of the Institute of Medical Biology, CAMS, with permit number [2018] 42, according to the national guidelines on animal work in China.

### Generation of Chimeric Mice by BM Reconstitution

BM was harvested from donor mice and transplanted into recipient mice as described previously (14). Briefly, 6- to 8-week-old recipient mice were lethally irradiated with two doses of 800 rad and 400 rad separated by an interval of 3 h. BM from donor mice was harvested from both the tibiae and femora under sterile conditions. The bones were flushed with RPMI 1640 (Thermo Fisher, Chengdu, China) containing 10% fetal bovine serum (FBS) (Life Technologies, MA, USA). After lysis of red cells with 0.15 M NH_4_Cl lysing solution, approximately 5 million cells were intravenously injected into recipient mice. The recipient mice were housed in a barrier facility (in individually ventilated cages with HEPA-filtered air) under pathogen-free conditions before and after BM transplantation. After BM transplantation, the mice were maintained on autoclaved water with antibiotics (5 mM sulfamethoxazole and 0.86 mM trimethoprim; Sigma-Aldrich, MD, USA) and were fed autoclaved food. These conditions were maintained for 5 weeks for recovery of the peripheral leukocytes before use. BM reconstitution was performed in 4 groups of mice: 1) WT mice reconstituted with BM from WT mice, 2) Cxcl5^-/-^ mice reconstituted with BM from WT mice, 3) WT mice reconstituted with BM from Cxcl5^-/-^ mice, and 4) Cxcl5^-/-^ mice reconstituted with BM from Cxcl5^-/-^ mice.

### Viral Loads and Titers

Mice were killed every 2 d following intranasal infection, and lung tissue samples were harvested. The samples were mechanically homogenized using a TGrinder instrument (Tiangen Biotechnologies, Beijing, China), and RNA was isolated using TRNzol Universal Reagent (DP424, Tiangen Biotechnologies, Beijing, China). The RNA concentration of each sample was determined by assessment of the UV 260/280 ratio using a NanoDrop 2000 (Thermo Fisher Scientific, MA, USA). A total of 100 ng of total RNA was reverse-transcribed and amplified using a One Step PrimeScript RT-PCR Kit (RR064A, Takara Biotechnologies, Dalian, China) on an Applied Biosystems 7500 Real-Time PCR System (Life Technologies, MA, USA). To determine the viral loads, the following primers and probe for the viral matrix protein gene (M gene) were used: 5’-AAGACCAATCCTGTCACCTCTGA-3’ (forward primer), 5’-CAAAGCGTCTACGCTGCAGTCC-3’ (reverse primer), and 5’-(FAM)-TTTGTGTTCACGCTCACCGT-(BHQ1)-3’ (probe). The M genes of the H1N1 viruses were cloned into the pMD18-T vector, and 10-fold serial dilutions of the clones were used to create a standard curve. The viral copy numbers were normalized to the masses of the original tissue samples and calculated based on the standard curve described above. The RNA was quantified in triplicate samples from each individual. Lung homogenates were centrifuged at 5000 rpm for 10 min at 4°C, and the supernatants were collected. The viral titers were determined as CCID_50_ titers by serial titration of the viruses in MDCK cells. The titers were calculated by the method of Reed and Muench.

### CyTOF

Mouse lung tissues were cut into small pieces and digested for 1 h at 37°C with rotation in RPMI 1640 medium (Thermo Fisher, Chengdu, China) containing 10% FBS (Life Technologies, MA, USA), collagenase type IV (350 U/ml), and deoxyribonuclease I (10 U/ml) (Sigma-Aldrich, MD, USA). The dissociated tissues were sequentially passed through 100 μm and 40 μm cell strainers and lysed with ACK lysis buffer. Mass cytometry analysis was conducted by Puluoting Health Technology (PLT Tech Co., Ltd., Zhejiang, China). Commercial antibodies were obtained from BioLegend (CA, USA), Thermo Fisher (Chengdu, China), BioXcell (NH, USA), R&D Systems (MN, USA) and BD Biosciences (NJ, USA) using the clones listed in **Supplemental Table 1**. Antibody labeling with the indicated metal tag was performed using a Maxpar Antibody Labeling Kit (Fluidigm, CA, USA). Conjugated antibodies were titrated to the optimal concentration before use. Cells were washed once with 1× PBS, stained with 100 μl of 250 nM cisplatin (Fluidigm, CA, USA) for 5 min on ice to exclude dead cells, and then incubated in Fc receptor blocking solution before staining with a surface antibody cocktail for 30 min on ice. The cells were washed twice with FACS buffer (1× PBS + 0.5% BSA) and fixed in 200 μl of intercalation solution (Maxpar Fix and Perm Buffer containing 250 nM 191/193Ir, Fluidigm, CA, USA) overnight. After fixation, the cells were washed once with FACS buffer, permeabilized (Thermo Fisher, MA, USA) and stained with an intracellular antibody cocktail for 30 min on ice. The cells were washed and resuspended in deionized water, added to 20% EQ beads (Fluidigm, CA, USA), and acquired on a mass cytometer (Fluidigm, CA, USA).

### Flow Cytometry

MLNs were harvested at the time of necropsy and filtered through 70μm cell strainer (BD Biosciences, NJ, USA) to collect single-cell suspensions. Lung tissues were cut into small pieces and digested 1 h at 37°C with rotation in RPMI 1640 medium (ThermoFisher, Chengdu, China) containing 10% FBS, Collagenase type IV (350 U/ml), and Deoxyribonuclase I (10U/ml) (Sigma-Aldrich, MD, USA). Dissociated tissues were sequentially passed through 100μm and 40μm cell strainer and lysed with ACK lysis buffer for preparation of single-cell suspensions. Cell number of each mouse lung was counted by hemocytometer. Cell surface stainings were performed using mouse BD Fc Block (clone 2.4G2, 553142), anti-mouse PerCP-CD45 (clone 30-F11, 557235), PE-Cy7-CD19 (clone 1D3, 552854), FITC-CD11c (clone HL3, 553801), BV421-B220 (clone RA3-6B2, 562922), PE-CD4 (clone H129.19, 553652), PE-CD138 (clone 281.2, 553714) from BD Biosciences (NJ, USA); anti-mouse PE-CD64 (clone X54-517.1, 139304), FITC-CD206 (clone C068C2, 141704), APC-CD274 (clone 10F.9G2, 124312), PE-Cy7-Ly6G (clone 1A8, 127618), APC-NK1.1 (clone PK136, 108710), PE-Cy7-CD274 (clone 10F.9G2, 124314), APC-CD8 (clone 53-5.8, 140410) from Biolegend (CA, USA); anti-mouse APC-CXCL13 (clone DS8CX13, 17-7981-82) and LIVE/DEAD™ Fixable Violet Dead cell Stain Kit (L34955) from ThermoFisher Scientific (MA, USA); APC-Flu tetramer NP366 (ASNENMETM) (H-2Db, HG08T7030) from HELIXGEN (Guangzhou, China). Intracellular stainings were performed after surface stainings and accomplished by fixation and permeabilization with BD Cytofix/Cytoperm solution (NJ, USA) according to the manufacturer’s protocol. Flow cytometric data were collected by CytoFLEX (Beckman Coulter, IN, USA) from single, live lung cells (**Figure S5**) and analyzed using the CytExpert version 2.3 (Beckman Coulter, IN, USA).

### Lung Histology, Immunohistochemical Analysis

Mice were sacrificed, and the lungs were slowly inflated by instilling 1 ml of 4% formaldehyde intratracheally. The trachea was ligated, and the lungs were fixed in formalin for 48 h at 4°C and embedded in paraffin. Lung tissue sections (5 mm) were stained with H&E according to routine procedures for histological analysis. The lung histological score was measured by a blinded pathologist on a 0-4 point scale according to the combined assessments of alveolar structure, inflammatory cell infiltration, aggregation in the alveolar space and septa, bronchiolitis, and lung edema. A score of 0 represented no damage, 1 represented mild damage, 2 represented moderate damage, 3 represented severe damage, and 4 represented very severe histological changes. An increment of 0.5 was used if the level of inflammation fell between two integers. In each tissue sample, three random areas were scored, and the mean value was calculated. The reported histology score is the median value from four mice. For immunohistochemical studies, lung sections were deparaffinized with xylene and rehydrated *via* a series of decreasing concentrations of ethanol. These sections were treated with 5% hydrogen peroxide in tris-buffered saline (pH 7.4) and then treated with serum to block nonspecific staining. The lung sections were then stained with a rabbit anti-mouse CD19 monoclonal antibody (ab245235, Abcam, Cambridge, UK) or a rat anti-mouse B220 monoclonal antibody (14-0452-82, Thermo Fisher, MA, USA) overnight at 4°C and subsequently incubated with a horseradish peroxidase-conjugated goat anti-rabbit secondary antibody (ab6721, Abcam, Cambridge, UK) or rabbit anti-rat secondary antibody (ab6734, Abcam, Cambridge, UK) for 30 min and counterstained with hematoxylin. Images were obtained using Slide Converter (3DHISTECH, Hungary).

### BALF/Plasma Sampling and Cytospin

Mice were first anesthetized, and plasma was collected using anti-coagulation with sodium citrate method as described previously (16). After the trachea of each mouse was exposed, the lungs were lavaged four times with 0.8 ml of cold sterile PBS, and the BALF was centrifuged at 1500 × *g* for 10 min at 4°C. Supernatants were collected and stored at -80°C for protein, chemokine, and cytokine detection. All of the leukocytes in the BALF were counted and resuspended at a concentration of 10^5^ cells/ml in PBS. For each sample, 200 μl of the solution was cytospun onto slides, and the cells were stained with a Wright–Giemsa Stain Kit (Nanjing Jiancheng Bioengineering, Jiangsu, China) for differential leukocyte counting under a microscope.

### *In Vitro* Macrophage Stimulation

Murine AMs were isolated from BALF as described previously (17). A total of 7 ×10^6^ AMs were obtained from 20 mice. The AMs were surface-stained with an anti-mouse PE-CD64 antibody (139304, BioLegend, CA, USA) and then positively selected and enriched with an EasySep™ Mouse PE Positive Selection Kit II (17666, STEMCELL Technologies, Vancouver, Canada). CD64+ AMs were grown at a density of 4 ×10^5^ cells/well in a 24-well plate using RPMI 1640 culture medium (Thermo Fisher, Chengdu, China) containing 10% FBS (Life Technologies, MA, USA) and were challenged with influenza H1N1 virus (multiplicity of infection, MOI=3). In some of the AM stimulation groups, recombinant proteins of the chemokines CXCL1 (453-KC), CXCL2 (452-M2), and CXCL5 (433-MC) (R&D Systems, MN, USA) were added to the culture medium (30 ng/ml each). The CXCR2 antagonist SB225002 from Selleck Chemicals (TX, USA) was used at 60 nM in the CXCR2 antagonism experiment. The PI3K inhibitor LY294002 (L9908, Sigma-Aldrich, MD, USA) and MEK inhibitor PD98059 (P215, Sigma-Aldrich, MD, USA) were used at 25 μM. After 10-20 h of incubation, the cells were harvested for detection of chemokine expression by reverse transcription quantitative polymerase chain reaction (RT-qPCR) assay, and the culture supernatants were collected for detection of chemokine protein levels by ELISA.

### *In Vivo* CXCL5 Administration and CXCR2 Blockade

An atraumatic orotracheal intubation method was used to intratracheally administer recombinant CXCL5 protein (18). Briefly, WT mice were anesthetized and orotracheally intubated with a 20-gauge angiocatheter with the guidance of an optical fiber source. Mice were subsequently placed in a vertical position suspended by their upper incisors. A polyethylene catheter was advanced into the trachea and used to instill either recombinant CXCL5 protein (1 μg per mouse per day) or sterile PBS as a control. In the CXCR2 antagonist experiments, a CXCR2 antagonist (SB225002, 2.5 μg/g per day) or control PBS was administered intraperitoneally.

### RT-qPCR Analysis

Macrophage cultures were collected, and total RNA was isolated using TRNzol Universal Reagent (DP424, Tiangen Biotechnologies, Beijing, China). RNA from the infected lung homogenates was isolated as described above. The primers used for RT-qPCR were as follows: CXCL5, 5’-GTTCCATCTCGCCATTCATGC-3’ (forward) and 5’-GCGGCTATGACTGAGGAAGG-3’ (reverse); CXCL1, 5’-ACTGCACCCAAACCGAAGTC-3’ (forward) and 5’-TGGGGACACCTTTTAGCATCTT-3’ (reverse); CXCL2, 5’-CCAACCACCAGGCTACAGG-3’ (forward) and 5’-GCGTCACACTCAAGCTCTG-3’ (reverse); CXCL13, 5’-ATATGTGTGAATCCTCGTGCCA-3’ (forward) and 5’-GGGAGTTGAAGACAGACTTTTGC-3’ (reverse); IFN-α, 5’-ACCCCTGCTATAACTATGACC-3’ (forward) and 5’-CTAACCACAGTGTAAAGGTGC-3’ (reverse); IFN-β, 5’-TTGCTCTCCTGTTGTGCTT-3’ (forward) and 5’-GCTGCTTCTTTGTAGGAATCCA-3’ (reverse); and GAPDH, 5’-ATGGAT GACGATATCGCTC-3’ (forward) and 5’-GATTCCATACCCAGGAAGG-3’ (reverse). RT-qPCR was carried out using the One Step SYBR PrimeScript RT-PCR Kit (RR066A, Takara Biotechnologies, Dalian, China) on an Applied Biosystems 7500 Real-Time PCR System (Life Technologies, MA, USA). The fold changes in the mRNA expression levels of these genes were calculated using the 2^-ΔΔCt^ method of relative quantification with GAPDH as the endogenous reference gene. All reactions were carried out in triplicate.

### ELISA

Protein samples from BALF, plasma, lung homogenates, and culture cell supernatants were collected as described above, and the concentrations of TNF-a (ab208348), IL-1b (ab197742), IL-6 (ab222503), and IFN-γ (ab100689) were determined using ELISA kits from Abcam (Cambridge, UK) in accordance with the manufacturer’s instructions. CXCL5 (MX000), CXCL1 (MKC00B), CXCL2 (MM200), CXCL12 (MCX120), and CXCL13 (MCX130) levels were determined using ELISA kits from R&D Systems (MN, USA) in accordance with the manufacturer’s instructions. For detection of virus-specific Igs, a 96-well High-Binding Flat-Bottom Microplate (Corning, USA) was coated with H1N1 virions in PBS at 4°C overnight. Coated plates were blocked with 0.2 ml of PBS containing 5% bovine serum albumin (BSA) at room temperature for 2 h. BALFs were 2-fold serial dilutions starting with a 1:1 dilution in virion binding assays. PBS was used as a negative control. After binding, the plates were washed 4 times with ice-cold PBS containing 0.1% Tween 20 (PBST) and the plates were incubated with rabbit/goat anti-mouse antibodies to IgG and IgA (ab6728, ab97235, Abcam, UK). The plate was then incubated with 100 µl of tetramethylbenzidine (TMB) ELISA Substrate (Solarbio, China) for 15 min at room temperature. Finally, the reaction was stopped with 100 µl of 0.5 M H_2_SO_4_. The absorbance was determined at 450 nm using a plate reader.

### Hemagglutination Inhibition (HI) Assay

BALF and serum samples were prepared for HI. Before the test, any nonspecific inhibitors of hemagglutination were removed by diluting the BALF or sera with a receptor-destroying enzyme (Denka Seiken, Tokyo, Japan) at a ratio of 1:5 and incubating the samples at 37°C overnight. The enzyme was inactivated by a 2 h incubation at 56°C followed by the addition of 0.1% sodium citrate. The HI assay was performed using the A/PR/8 strain with 1% chicken erythrocytes in V-bottom 96-well microtiter plates.

### Western Blot Assay

Protein extracts were prepared from cultured AMs using RIPA lysis buffer (R0010, Solarbio, Beijing, China). Proteins were separated using sodium dodecyl sulfate-polyacrylamide gel electrophoresis (SDS-PAGE) and transferred to polyvinylidene fluoride (PVDF) membranes. The membranes were stained with a rabbit anti-mouse CXCR2 polyclonal antibody (ab65968, Abcam, UK) and a goat anti-rabbit IgG H&L (HRP) polyclonal antibody (ab6721, Abcam, UK). The proteins were detected using SuperSignal West Pico PLUS Chemiluminescent Substrate (Thermo Fisher, MA, USA).

### Immunofluorescence Assay

Lung tissue sections in paraffin were subjected to deparaffinization in xylene and rehydration in a graded series of ethanol followed by rinsing in double-distilled water. The rehydrated tissue sections were blocked for 2 h in 10% FBS. The slides were then incubated with an Alexa Fluor 647-conjugated rat anti-mouse CD45R (B220) monoclonal antibody (557683, BD Biosciences, NJ, USA) and an eFluor 570-conjugated rat anti-mouse CD4 monoclonal antibody (41-9766-82, Thermo Fisher, MA, USA) for 2 h at room temperature. The cell nuclei were stained with DAPI (ab104139, Abcam, USA), and images were captured using a Leica TCS SP8 laser confocal microscope.

### Transcriptome Analysis of Lung Tissues

Transcriptome analysis was conducted by HaploX Genomics Center (HGC, Shenzheng, China). Briefly, total RNA from the lungs of WT and CXCL5^-/-^ infected mice (n=3) was extracted using TRIzol Reagent (DP424, Tiangen Biotechnologies, Beijing, China). The RNA concentration was measured using a Qubit^®^ RNA Assay Kit in a Qubit^®^ 2.0 Fluorometer (Life Technologies, CA, USA), and integrity was assessed using the RNA Nano 6000 Assay Kit of the Bioanalyzer 2100 system (Agilent Technologies, CA, USA). Sequencing libraries were generated by polymerase chain reaction (PCR) amplification and purified from total RNA using poly-T oligo-attached magnetic beads. Fragmentation was carried out using divalent cations under elevated temperature in NEBNext First Strand Synthesis Reaction Buffer (5×). To preferentially select cDNA fragments 250~300 bp in length, the library fragments were purified with an AMPure XP system (Beckman Coulter, Beverly, USA). Then, 3 µl of USER Enzyme (NEB, MA, USA) was used with size-selected, adaptor-ligated cDNA at 37°C for 15 min followed by 5 min at 95°C before PCR. Then, PCR was performed with Phusion High-Fidelity DNA polymerase, universal PCR primers and Index (X) Primer (Thermo Fisher, MA, USA). Finally, the PCR products were purified (AMPure XP system, Beckman Coulter, IN, USA), and library quality was assessed on the Agilent Bioanalyzer 2100 system (Agilent Technologies, CA, USA). The libraries were pooled and sequenced (paired-end 50 bp) on an Illumina PE150 platform to an average depth of 60 million reads per sample, and the sequences were assigned to their samples of origin according to the indices used. For data analysis, the raw sequencing reads were processed by FastQC (https://www.bioinformatics.babraham.ac.uk/projects/fastqc/) and aligned by STAR33 to the mouse Ensembl genome (Ensembl, GRCm38.p5) with Ensembl annotations (Mus_musculus.GRCm38.83.gtf). Expression matrices were generated using RSEM v1.3.0. Differential expression analysis was performed for protein-coding genes using the R/Bioconductor package DESeq2 v1.16.1 with standard parameters. We applied the DESeq2 algorithm to filter the differentially expressed genes after significance analysis and FDR analysis under the following criteria: i) log_2_FC > 1 and ii) P value < 0.05. The Database for Annotation, Visualization and Integrated Discovery (DAVID) (http://david.abcc.ncifcrf.gov/), which utilizes the GO database to identify the biological processes, molecular functions, and cellular components associated with common differentially expressed genes, was applied in the current study.

### Statistical Analysis

We performed all the statistical analyses with GraphPad Prism software (version 8, CA, USA) and Microsoft Excel for Windows (version 2010, WA, USA). The data that were obtained from all experiments are presented as the mean ± SD. Means were compared between two groups at a single time point with Student’s *t*-test, and P<0.05 was considered to indicate statistical significance.

## Results

### Respiratory H1N1 Infection-Induced Production of CXCL5 Protein in Infected Lungs

**Figure 1:**
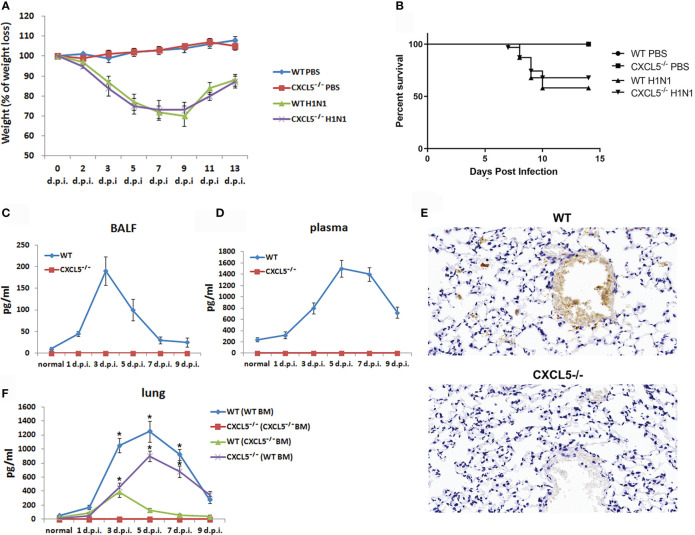
Respiratory H1N1 infection mouse model and CXCL5 protein production in infected lungs. WT mice, CXCL5^-/-^ mice and BM reconstitution mice were infected with H1N1 virus (1000 CCID_50_), and PBS instillation was used as a control. **(A)** The body weight loss of the mice (n=16) was monitored until 2 weeks post infection. **(B)** Kaplan-Meier survival curve for the infected mice (n=31). **(C)** CXCL5 levels in the BALF of the infected mice were measured by ELISA (n=5). **(D)** CXCL5 levels in the plasma of the infected mice were measured by ELISA (n=5). **(E)** Lung sections from infected WT and CXCL5-/- mice were stained at 3 d.p.i. with anti-CXCL5 antibody and hematoxylin. **(F)** CXCL5 levels in the lung homogenates of the BM reconstitution mice were measured by ELISA (n=4). WT(WT BM): WT recipient mice reconstituted with BM from WT donor mice; WT(CXCL5^-/-^ BM): WT recipient mice reconstituted with BM from CXCL5^-/-^ donor mice; CXCL5^-/-^(CXCL5^-/-^ BM): CXCL5^-/-^ recipient mice reconstituted with BM from CXCL5^-/-^ donor mice; CXCL5^-/-^(WT BM): CXCL5^-/-^ recipient mice reconstituted with BM from WT donor mice. The error bars represent the SDs. ^*^P < 0.05 based on Student’s *t*-test.

Wild-type (WT) and CXCL5^-/-^ mice intranasally infected with a lethal dose of H1N1 (1000 50% cell culture infective dose, CCID_50_) were monitored for body weight changes and survival. As shown in **Figure 1A**, WT and CXCL5^-/-^ mice gradually lost weight after infection, and the greatest weight loss (20%-30%) was observed at 7 to 9 days post infection (d.p.i.). Approximately half of the WT infected mice died from 7-10 d.p.i. (survival rate, 56%), while approximately 69% of infected CXCL5^-/-^ mice survived beyond 9 d.p.i. (**Figure 1B**). The mice recovered by 9 d.p.i. as indicated by body weight, and no deaths occurred after 10 d.p.i. in either mouse group (**Figures 1A, B** **)**. To determine whether CXCL5 was correlated with influenza challenge, CXCL5 protein levels were measured in local lung bronchoalveolar lavage fluid (BALF) and systemic blood circulation. Upon H1N1 infection, the level of CXCL5 protein in BALF increased rapidly after 1 d.p.i., peaked at 3 d.p.i. (at approximately 200 pg/ml) and then decreased rapidly to the baseline value at 7 d.p.i. (**Figure 1C**). Meanwhile, the plasma protein level of CXCL5 began to increase after 1 d.p.i., remained high from 5-7 d.p.i. (at approximately 1500 pg/ml) and then decreased (**Figure 1D**). Immuno-histochemical analysis of the infected lungs confirmed the expression of CXCL5 in the lung parenchyma and the lung capillaries (**Figure 1E**). These data suggest that respiratory H1N1 infection induces increases in CXCL5 levels locally in infected lungs and in the blood circulation. During lung inflammation, CXCL5 can be produced by lung resident epithelial cells and released by activated platelets (13, 14). To further determine whether the upregulation of CXCL5 in infected lungs is due to expression by lung resident cells and/or release by activated platelets in the pulmonary capillary circulation, chimeric mice were generated using bone marrow (BM) reconstitution. WT mice reconstituted with BM from WT or CXCL5^-/-^ mice were designated WT (WT BM) and WT (CXCL5^-/-^ BM) mice, respectively, and CXCL5^-/-^ mice reconstituted with BM from CXCL5^-/-^ or WT mice were designated CXCL5^-/-^ (CXCL5^-/-^ BM) and CXCL5^-/-^ (WT BM) mice, respectively. Detection of the lung tissue CXCL5 levels of WT (WT BM) mice showed that the levels increased after 1 d.p.i., maintained peak values between 3 and 7 d.p.i. (at approximately ≥ 1000 pg/ml) and then decreased (**Figure 1F**). In contrast, CXCL5 levels in WT (CXCL5^-/-^ BM) mice increased from 1 d.p.i. to 3 d.p.i. (peak value: approximately 400 pg/ml) and then rapidly decreased after 3 d.p.i. (**Figure 1F**). Moreover, CXCL5^-/-^(WT BM) mice regained expression of CXCL5 post infection, and the dynamic change was similar to that in WT (WT BM) mice (**Figure 1F**). Together, these data suggested that reconstitution of WT mice with the hematopoietic compartment of CXCL5^-/-^ mice markedly decreased the levels of CXCL5 in infected lungs post infection, while reconstitution of CXCL5^-/-^ mice with the hematopoietic compartment of WT mice resulted in CXCL5 production. Considering that WT (CXCL5^-/-^ BM) mice still expressed CXCL5 in the infected lungs after infection, the data demonstrate that influenza infection induces both CXCL5 production by lung tissue cells and CXCL5 release by blood-activated platelets, and the amount of CXCL5 protein released in the blood is greater than that produced by lung tissue cells in influenza virus-infected lungs.

### CXCL5 Deficiency Led to Reduced Lung Inflammation in the Early Innate Immune Response and Altered Viral Clearance in the Infected Lungs

**Figure 2:**
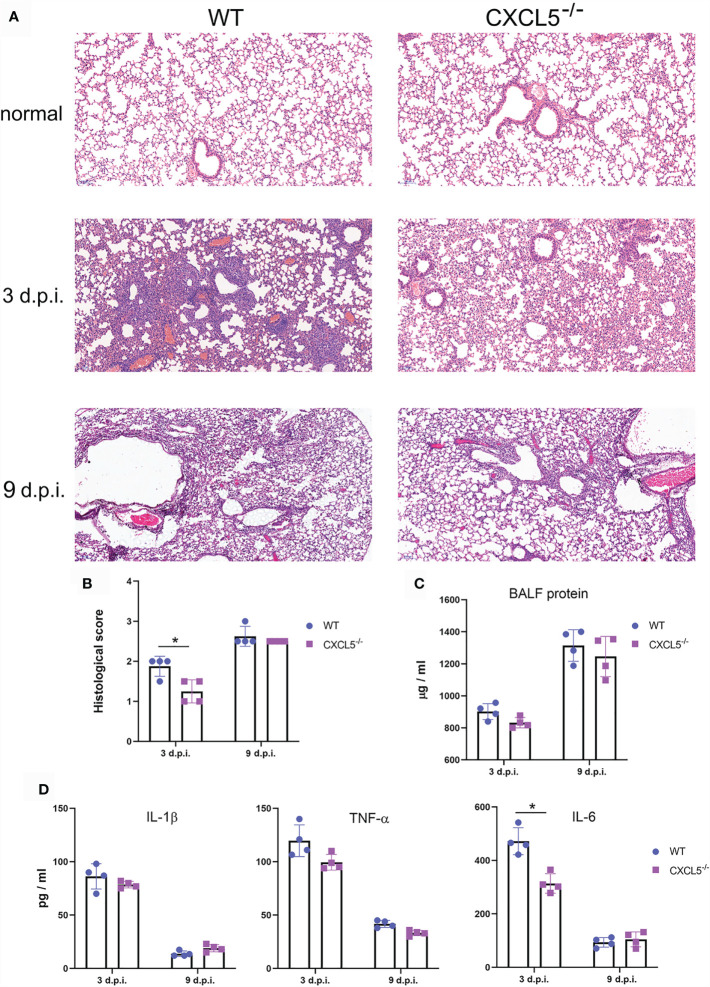
Comparison of lung pathology and inflammation between WT and CXCL5^-/-^ infected mice. **(A)** Histopathology of H&E–stained lung sections from WT and CXCL5^-/-^ mice 3 and 9 d.p.i. **(B)** Lung histological scores were assessed for 4 mice in each group by a blinded veterinary pathologist as described in the MATERIALS AND METHODS section. **(C)** The total protein concentrations in the BALF of infected mouse lungs were determined at 3 and 9 d.p.i. **(D)** The levels of the inflammatory cytokines TNF-a, IL-1β, and IL-6 were measured by ELISA. The error bars represent the SDs of 4 samples. ^*^P < 0.05 based on Student’s *t*-test.

Lung inflammation was assessed and compared between WT mice and CXCL5^-/-^ mice during the early and late stages of infection. Hematoxylin and eosin (H&E) staining of lung tissue revealed that both mouse groups developed lung inflammation during the early innate immune response (3 d.p.i.), with symptoms including inflammatory cell infiltration into the lung interstitium and thickening of the bronchial epithelium (**Figure 2A**). Compared to CXCL5^-/-^ infected mice, WT infected mice presented more inflammatory cell aggregation in the bronchial walls and alveolar spaces (**Figure 2A**). Histological scoring indicated that CXCL5^-/-^ mice had less severe lung pathology than WT mice during the early infection stage (**Figure 2B**). During the late infection stage (9 d.p.i.), lung inflammation gradually regressed in the surviving mice of both groups; the alveolar structure tended to be complete, but a certain number of inflammatory cells aggregated around the bronchial epithelium (**Figure 2B**). No significant differences in pathological changes were observed between the two groups during the late infection stage (9 d.p.i.) (**Figure 2B**). In addition, detection of BALF total protein concentrations and inflammatory cytokine levels also showed inflammatory development in the infected lungs upon influenza infection in both mouse groups, and no significant differences in BALF total protein concentrations, TNF-α levels, or IL-1β levels were observed at 3 d.p.i. or 9 d.p.i. (**Figures 2C, D** **)**. IL-6 levels in CXCL5^-/-^ mice were significantly lower than those in WT mice at 3 d.p.i. but similar to those in WT mice at 9 d.p.i. (**Figure 2D**).

**Figure 3:**
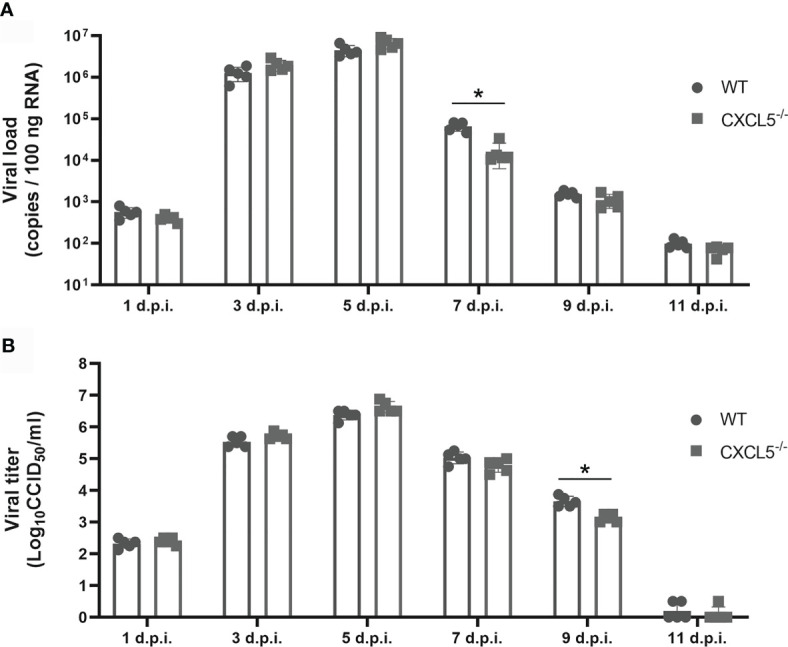
Viral loads and viral titers after influenza infection. Lungs were harvested from H1N1-infected WT and CXCL5^-/-^ mice, and homogenates were prepared for analysis of viral titers and loads. **(A)** Viral loads were determined based on the number of influenza M gene RNA copies detected by RT-qPCR at different d.p.i. **(B)** The titers of infectious viruses were determined using a standard CCID_50_ assay at different d.p.i. The error bars represent the SDs of 5 samples. ^*^P < 0.05 based on Student’s *t*-test.

Viral replication and proliferation in the lung tissues of infected mice were evaluated by viral load and viral titer tests. As shown in **Figure 3A**, viral loads reached peak levels in both WT and CXCL5^-/-^ mice between 3 and 5 d.p.i. but began to decline after 5 d.p.i. Of note, the viral loads of CXCL5^-/-^ mice were significantly lower than those of WT mice (approximately 10 times lower) at 7 d.p.i. Exhibiting the same trend as the viral loads, the viral titers also increased to high levels from 3-5 d.p.i. before beginning to decline, and the virus was cleared to 1 CCID_50_ by 11 d.p.i. (**Figure 3B**). At 9 d.p.i., the viral titers of CXCL5^-/-^ mice were significantly lower than those of WT mice (**Figure 3B**). The data on viral replication and proliferation suggest that CXCL5 deficiency facilitates pulmonary viral clearance to a certain extent during the late stage of infection.

### CXCL5 Deficiency Impaired Pulmonary Neutrophil Infiltration but Contributed to Accumulation of B Lymphocytes in Infected Lungs After Influenza Infection

**Figure 4:**
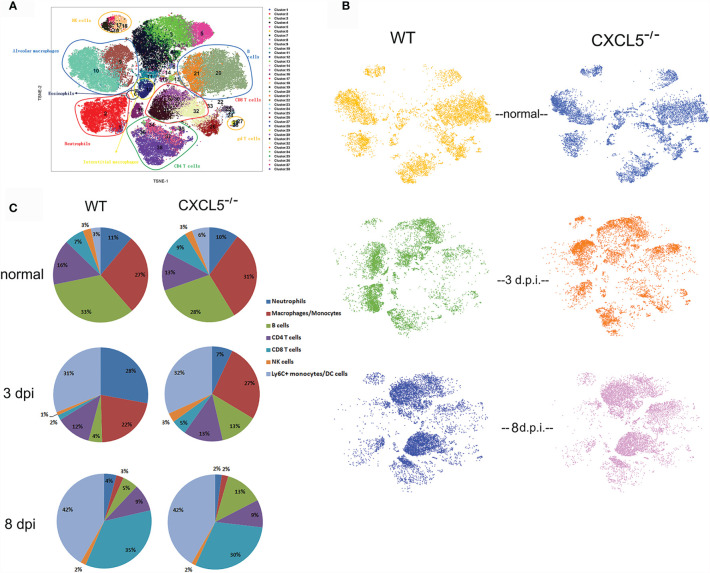
CyTOF analysis and comparison of leukocyte compositions in WT and CXCL5^-/-^ mouse lungs before and after influenza infection. **(A)** tSNE analysis of CD45+ single-cell data from the mouse lung tissues. The cells were plotted and color-coded according to 38 clusters using the X-shift clustering algorithm. The detailed characterization of each cluster is shown in **Supplemental Figure 2**. The different cell subsets were divided by their surface marker expression patterns in normal mouse lungs as described previously (18). **(B)** tSNE analysis of the leukocyte compositions of lung tissues derived from WT and CXCL5^-/-^ mice (n=1) before and after (at 3 d.p.i. and 8 d.p.i.) influenza infection. **(C)** The cell frequencies of the indicated major leukocyte subsets are illustrated for the lungs of WT and CXCL5^-/-^ mice (n=1).

**Figure 5:**
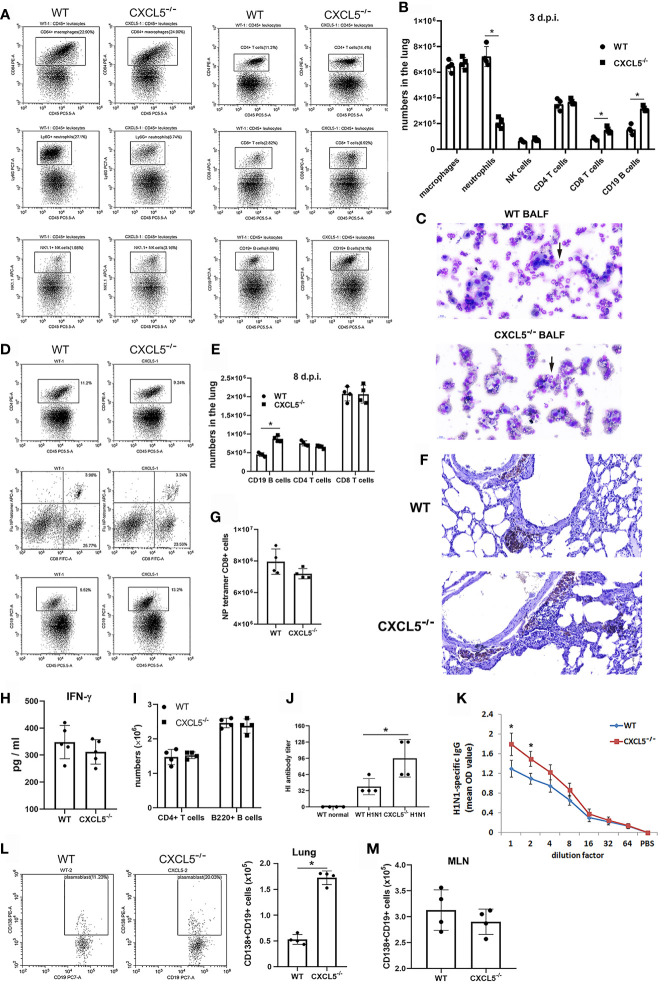
Pulmonary innate and adaptive immune responses induced by influenza infection in WT and CXCL5^-/-^ mice. **(A)** Single lung cells were collected from WT and CXCL5^-/-^ mice at 3 d.p.i. and analyzed by flow cytometry to detect macrophages (CD64+CD45+), neutrophils (Ly6G+CD45+), NK cells (NK1.1+CD45+), CD4 T cells (CD4+CD45+), CD8 T cells (CD8+CD45+), and CD19 B cells (CD19+CD45+). **(B)** Numbers of different leukocyte subsets among pulmonary CD45+ leukocytes at 3 d.p.i. (n=4). **(C)** Microscopic view of BALF leukocytes from WT and CXCL5^-/-^ mice collected at 3 d.p.i. and cytospun. Black arrows, neutrophils (×400 magnification). **(D)** Single lung cells were collected from WT and CXCL5^-/-^ mice at 8 d.p.i. and analyzed by flow cytometry to detect CD4 T cells (CD4+CD45+), CD8 T cells (CD8+CD45+), CD19 B cells (CD19+CD45+), and influenza-specific CD8 T cells (APC-conjugated class I tetramers loaded with the influenza virus NP peptide ASNENMETM). **(E)** Numbers of different leukocyte subsets among pulmonary CD45+ leukocytes at 8 d.p.i. (n=4). **(F)** Lung sections from WT and CXCL5^-/-^ mice were stained at 3 d.p.i. with an anti-CD19 antibody and hematoxylin (×200 magnification). **(G)** Numbers of NP tetramer-positive CD8+ T cells among pulmonary CD45+ leukocytes at 8 d.p.i. (n=4). **(H)** The levels of IFN-γ in BALF from WT and CXCL5^-/-^ mice at 8 d.p.i. were determined by ELISA (n=5). **(I)** The total numbers of CD4+ T cells and B220+ B cells in the MLNs at 8 d.p.i. were determined by cell counting together with flow cytometry (n=4). **(J)** The HI titers of the BALF samples from WT and CXCL5^-/-^ mice at 8 d.p.i. against H1N1 virus were evaluated (n=4). **(K)** Measurements of influenza-specific total IgG in the BALF of WT and CXCL5^-/-^ mice infected with the H1N1 virus at 8 d.p.i. (n = 5). **(L)** The numbers of plasmablasts among pulmonary B cells were detected using anti-CD19 and anti-CD138 antibodies by flow cytometry of samples from WT and CXCL5^-/-^ mice at 8 d.p.i. (n=4). **(M)** The total numbers of CD138+CD19+ B cells in the MLNs at 8 d.p.i. were determined by cell counting together with flow cytometry (n=4). The error bars represent the SDs. ^*^P < 0.05 based on Student’s *t*-test.

**Figure 6:**
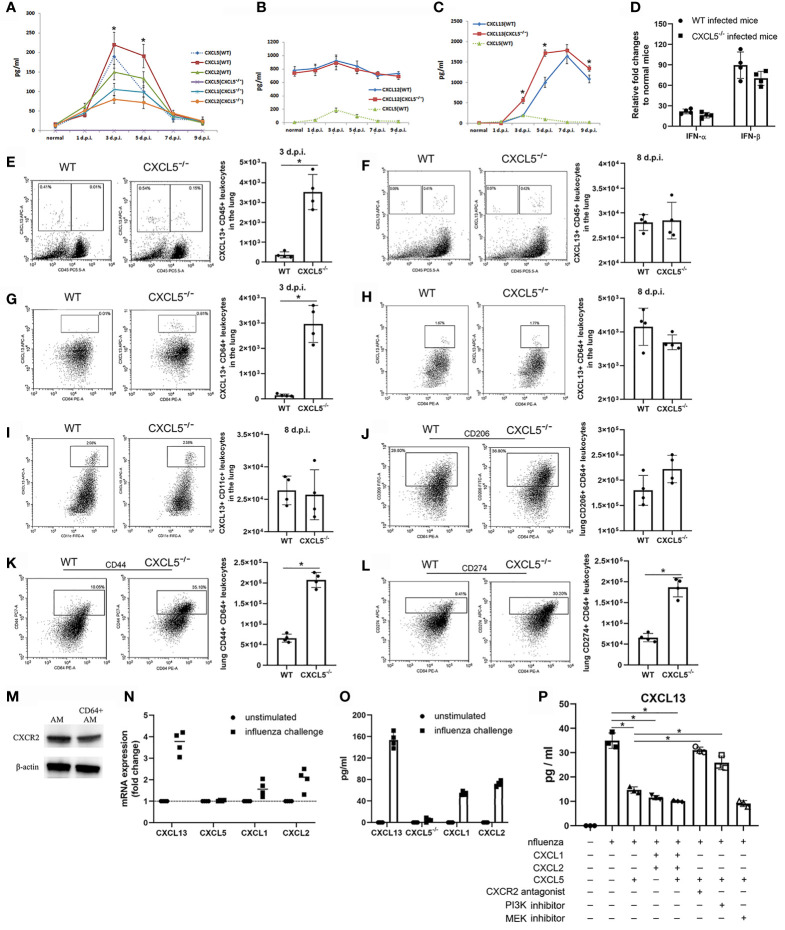
CXCL5 regulated CXCL13 expression in pulmonary CD64+ macrophages/monocytes *via* the CXCL1/2/5-CXCR2 signaling pathway. **(A–C)** The protein levels of CXCL1/2 **(A)**, CXCL12 **(B)**, and CXCL13 **(C)** in the BALF of infected mice were measured by ELISA, and the level of CXCL5 was used as a reference (n=5). **(D)** RT-qPCR for IFN-α and IFN-β mRNA was performed in lung homogenates from WT and CXCL5^-/-^ mice before and after (3 d.p.i.) influenza infection. The fold changes in the mRNA expression levels of these genes were calculated using the 2^-ΔΔCt^ method of relative quantification with GAPDH as the endogenous reference gene. The relative fold changes in IFN-α and IFN-β expression in infected mice compared to normal mice are presented (n=4). **(E, F)** Percentages and numbers of CXCL13-expressing cells among single lung cells from WT and CXCL5^-/-^ mice at 3 d.p.i. **(E)** and 8 d.p.i. **(F)** as assessed by intracellular staining of CXCL13 protein (APC-CXCL13) and flow cytometry (n=4). **(G, H)** Percentages and numbers of CXCL13-expressing cells among pulmonary CD64+ cells from WT and CXCL5^-/-^ mice at 3 d.p.i. **(G)** and 8 d.p.i. **(H)** as assessed by intracellular staining of CXCL13 protein (APC-CXCL13) and flow cytometry (n=4). **(I)** Percentages and numbers of CXCL13-expressing cells among pulmonary CD11c+ cells from WT and CXCL5^-/-^ mice at 8 d.p.i. as assessed by intracellular staining of CXCL13 protein (APC-CXCL13) and flow cytometry (n=4). **(J–L)** Percentages and numbers of CD206 **(J)**, CD44 **(K)**, and CD274 **(L)** surface markers among pulmonary CD64+ cells from WT and CXCL5-/- mice at 3 d.p.i. (n=4). **(M)** Detection of the expression of mouse CXCR2 on total AMs and CD64+ AMs by western blotting. **(N)** RT-qPCR was performed on cell lysates of cultured CD64+ macrophages after 10 h of influenza challenge or unstimulated (control) conditions, and the fold changes were calculated as described in the MATERIALS AND METHODS section (n=4). **(O)** Expression levels of the indicated chemokines from supernatants of cultured CD64+ macrophages after 20 h of influenza challenge or unstimulated (control) conditions as determined by ELISA (n=4). **(P)** ELISA was used to determine the expression levels of CXCL13 protein in supernatants of cultured CD64+ macrophages after 20 h of influenza challenge and treatment with the indicated recombinant chemokine proteins, the CXCR2 antagonist SB225002, the PI3K inhibitor LY294002, and the MEK inhibitor PD98059 (n=3). The error bars represent the SDs. ^*^P < 0.05 based on Student’s *t*-test.

In order to understand the lung immune landscape changes after H1N1 infection and to dissect the differences in lung immunity between WT and CXCL5^-/-^ infected mice, mass cytometry by time-of-flight (CyTOF) was conducted to compare the lung leukocyte compositions during the normal stage, early infection stage (3 d.p.i.), and late infection stage (8 d.p.i.) between WT and CXCL5^-/-^ mice. Lung cells from infected and uninfected mice were stained with a panel of 42 markers (**Supplementary Table 1**) and analyzed using CyTOF. Single-cell CD45+ leukocytes (**Supplementary Figure 1A**) were selected, and analysis was performed using t-distributed stochastic neighbor embedding (tSNE) in conjunction with the X-shift clustering algorithm. A total of 38 clusters were identified with shared surface marker expression characteristics (**Supplementary Figure 2**). Major immune cell subsets, such as neutrophils, monocytes, alveolar macrophages (AMs), interstitial macrophages, eosinophils, NK cells, B cells, CD4+ T cells, CD8+ T cells, and γδ T cells, were characterized based on the expression patterns of different leukocytes in normal mouse lungs as described previously (**Figure 4A** and **Supplementary Figure 2**) (19). Based on CyTOF results, lymphocytes (B and T cells, approximately 50%), macrophages/monocytes (approximately 30%) and neutrophils (approximately 10%) were the main categories of leukocytes in normal lungs in both CXCL5^-/-^ and WT mice (**Figures 4B, C** **)**. While macrophages reside in the lung alveoli and interstitium, lymphocytes and neutrophils arise primarily from the pulmonary capillary circulation (20). Upon infection, the leukocyte compositions in infected lungs substantially changed. Specifically, large numbers of neutrophils, monocytes, macrophages, and DCs accumulated in the lungs (total, approximately 80%) during the early infection stage (3 d.p.i.). During the late stage of infection (8 d.p.i.), the main types of leukocytes in the infected lungs changed to lymphocytes (49%) and DCs/monocytes (42%) (**Figures 4B, C** **)**. Neutrophil infiltration in infected lungs is a hallmark of the innate immune response, and significantly lower neutrophil percentages in infected lungs were observed in CXCL5^-/-^ mice than in WT mice at 3 d.p.i. (**Figures 4B, C** **)**. In contrast, the percentages of B cells, CD8+ T cells, NK cells, and macrophages were relatively increased at this time (**Figures 4B, C** **)**. During the late infection stage (8 d.p.i.), a notable difference in the B cell percentage was observed in CXCL5^-/-^ mice (13%) compared to WT mice (5%) (**Figures 4B, C** **)**. Data from CyTOF indicated that the phenotypes of innate and adaptive leukocyte compositions differed between WT and CXCL5^-/-^ infected mice. We further performed histochemical detection and flow cytometry analysis to confirm the leukocyte compositions in the infected lungs of WT and CXCL5^-/-^ mice. As shown in **Figures 5A, B**, the pulmonary neutrophil number was significantly lower in CXCL5^-/-^ mice than in WT mice, and the numbers of CD8+ T cells and CD19+ B cells were higher in CXCL5^-/-^ mice than in WT mice at 3 d.p.i., consistent with the data from CyTOF. BALF cytospin also demonstrated that neutrophil recruitment in infected lungs was significantly lower in CXCL5^-/-^ mice than in WT mice during the early infection stage (**Figure 6C**). During the late infection stage (8 d.p.i.), among different lymphocyte subsets, only the CD19+ B cell subset exhibited a significantly higher number in CXCL5^-/-^ mice than in WT mice (**Figures 5D, E** **)**. The results indicated that increased B cell accumulation occurred in the infected lungs of CXCL5^-/-^ mice after influenza infection (3-8 d.p.i.). To confirm the accumulation of B cells in lung capillaries or lung parenchyma, immunohistochemistry against CD19+ B cells was conducted. The results revealed that B cells were recruited into the lung tissues and accumulated in the parenchyma as soon as at 3 d.p.i. (**Figure 5F**). Together, the above data suggest that the major effects of CXCL5 deficiency on the pulmonary influenza-induced immune response are to impair pulmonary neutrophil infiltration in the infected lungs at the innate immunity stage and to promote B lymphocyte accumulation in infected lungs after influenza infection.

### CXCL5 Deficiency Enhanced the Pulmonary Anti-H1N1 Antibody Response

The adaptive immune response is critical for viral clearance upon pulmonary influenza infection, and T, B cell immune responses were evaluated at 8 d.p.i. The numbers of pulmonary CD4+ T cells and CD8+ T cells were comparable between WT and CXCL5^-/-^ mice at 8 d.p.i. (**Figures 5D, E** **)**. Staining of CD8+ T cells with major histocompatibility complex class I tetramers loaded with influenza virus NP peptide showed no significant difference in virus-specific CD8+ T cell number between WT and CXCL5^-/-^ mice at 8 d.p.i (**Figures 5D, G** **)**. In addition, the levels of BALF IFN-γ, normally associated with the Th1 cell antiviral response, were not significantly different between WT and CXCL5^-/-^ mice at 8 d.p.i. (**Figure 5H**). The data do not indicate an elevated T cell immune response in CXCL5^-/-^ infected mice. On the other hand, the B cell numbers in infected lungs were significantly higher in CXCL5^-/-^ mice than in WT mice at 8 d.p.i. (**Figures 5D, E** **)**, while the numbers of B220+ B cells and CD4+ T cells in the lung-draining mediastinal lymph nodes (MLNs) were comparable between the two groups (**Figure 5I**). An influenza hemagglutination inhibition assay revealed that total H1N1-specific antibody levels in lung BALF but not in circulating system were significantly higher in CXCL5^-/-^ mice than in WT mice at 8 d.p.i. (**Figure 5J** and **Supplementary Figure 3A**). Moreover, detection of H1N1-specific IgG and IgA antibodies using ELISA also showed that virus-specific IgG and IgA levels were markedly higher in lung BALF of CXCL5^-/-^ mice than in WT mice (**Figure 5K** and **Supplementary Figure 3B**). Thus, these data suggest that CXCL5 deficiency contributes to an enhanced virus-specific antibody response in infected lungs during the adaptive immunity stage. To further assess whether the elevated antibody levels in CXCL5^-/-^ infected mice were caused by antibodies from MLN activated B cells or from accumulated pulmonary B cells, flow cytometry analysis of plasmablast B cells was conducted. The data revealed that the number of pulmonary CD19+CD138+ plasmablasts was significantly higher in CXCL5^-/-^ infected mice than in WT infected mice (**Figure 6L**), while the numbers of MLN CD19+CD138+ plasmablasts were comparable between WT and CXCL5^-/-^ infected mice (**Figure 6M**). The results suggest that the elevated pulmonary antiviral antibody levels in the infected lungs of CXCL5^-/-^ mice are attributable to the local B cell immune response.

### CXCL5-CXCR2 Signaling Regulated CXCL13 Expression in Pulmonary CD64+ Macrophages/Monocytes Upon H1N1 Infection

As a major neutrophil chemoattractant, CXCL5 shares the same CXCR2 signaling pathway with CXCL1 and CXCL2 to induce neutrophil recruitment and mediate inflammation (6, 8). However, a previous study by Mei et al. has suggested that CXCL5 negatively regulates neutrophil influx to the lungs in severe *E. coli* pneumonia by influencing the concentrations of CXCL1 and CXCL2 in blood *via* scavenging by DARC (14). Neutrophil infiltration in infected lungs during the early infection stage of respiratory H1N1 infection was significantly decreased in the CXCL5^-/-^ mouse model (**Figures 5A, C** **)**, which suggests that CXCL5 positively regulates neutrophil influx to infected lungs during the innate immunity stage in the H1N1 infection model. In addition, both CXCL1 and CXCL2 levels in BALF were dramatically decreased from 3-5 d.p.i. in CXCL5^-/-^ mice compared to WT mice (**Figure 6A**), which also contributed to the reductions in neutrophil numbers in the infected lungs of CXCL5^-/-^ mice.

B cells are usually found in lymphoid tissue and the blood circulation, where they produce antibodies to exhibit immune function. The accumulation of B cells in the infected lungs of CXCL5^-/-^ mice was possibly due to enhanced B cell recruitment in the infected lungs. CXCL12 and CXCL13 are two well-documented B cell chemoattractants (20, 21), and detection of CXCL12 and CXCL13 levels in BALF after viral infection revealed that CXCL13 expression at 3-5 d.p.i. was significantly higher in CXCL5^-/-^ mice than in WT mice, while CXCL12 expression was comparable between the two mouse groups (**Figures 6B, C** **)**. In CXCL5^-/-^ mice, CXCL13 levels rapidly increased to achieve high levels (approximately 1800 pg/ml) by 5 d.p.i. and remained relatively high from 5-9 d.p.i. (≥1400 pg/ml) (**Figure 6C**). CXCL13 levels in WT mice increased to peak at 7 d.p.i. and then decreased to approximately 1100 pg/ml by 9 d.p.i., reaching levels significantly lower than those in CXCL5^-/-^ mice (**Figure 6C**). Of note, CXCL13 expression increased with decreasing CXCL5 expression (**Figure 6C**). CXCL13 was originally found to be expressed by follicular DCs and B cells in the second lymphoid organ and was recently found to be expressed in lung fibroblasts, lung AMs, and peritoneal macrophages (20–26). Influenza infection induces pulmonary CXCL13 expression by type I IFN-activated lung fibroblasts (20); however, the levels of pulmonary IFN-α/β were comparable between WT and CXCL5^-/-^ infected mice (**Figure 6D**). To further determine which subsets of lung cells contributed to increasing the expression of CXCL13 in the infected lungs of CXCL5^-/-^ mice during the early influenza infection stage, single lung cells were analyzed using flow cytometry and intracellularly stained with a fluorescent anti-CXCL13 antibody. As shown in **Figure 6E**, CXCL13-expressing cells in CXCL5^-/-^ mice included both lung tissue cells and lung leukocytes, while almost all CXCL13-expressing cells in WT mice were lung tissue cells at 3 d.p.i., which suggested that the increased expression of CXCL13 during the early infection stage was attributable to pulmonary lymphocytes. At 8 d.p.i., however, CXCL13 was expressed mainly by pulmonary leukocytes in both WT and CXCL5^-/-^ mice (no significant difference), while a small amount of CXCL13 was expressed by tissue cells (**Figure 6F**). Further gating on pulmonary lymphocyte subsets indicated that CD64+ macrophages/monocytes and CD11c+ cells were responsible for the expression of CXCL13 during the early (3 d.p.i.) and late (8 d.p.i.) infection stages respectively (**Figures 6G, I** **)**. In addition, CD64+ macrophages/monocytes also expressed CXCL13 at 8 d.p.i. (**Figure 6H**). As multidimensional cells, macrophages, upon activation by exogenous stimuli, display different phenotypes and participate in diverse functional programs; the process by which these different phenotypes are acquired is called macrophage polarization (27). CXCL13 is expressed by M2-phenotype macrophage after macrophage activation (28, 29). We thus further detected M2 macrophages using the marker CD206, and the data showed that CD206 expression on CD64+ macrophages/monocytes was higher in CXCL5^-/-^ mice than in WT mice, but the difference was not statistically significant (**Figure 6J**). Examination of the CyTOF leukocyte clusters of AMs from 3 d.p.i. revealed that AMs of CXCL5^-/-^ mice were concentrated in cluster 9, while AMs of WT mice were mixed between clusters 9 and 10 (**Figure 4A** and **Supplementary Figure 2**). At 3 d.p.i., the phenotypes of AMs were different between CXCL5^-/-^ and WT infected mice (**Supplementary Figure 2**). We further performed flow cytometry to analyze the surface expression of different markers on CD64+ macrophages/monocytes in cluster 9 and cluster 10. The expression of two surface markers, CD44 and CD274, on CD64+ macrophages/monocytes was significantly higher in CXCL5^-/-^ mice than in WT mice at 3 d.p.i. (**Figures 6K, L** **)**. Together, the data suggest that CXCL5 deficiency induced CXCL13 expression in pulmonary CD64+ macrophages/monocytes in influenza-infected lungs beginning in the early innate immunity stage.

The finding that loss of CXCL5 signaling triggers CXCL13 expression in CD64+ macrophages/monocytes in influenza-infected mouse lungs suggests that CXCL5 negatively regulates CXCL13 expression in macrophages. As mentioned above, CXCL5 exerts its function *via* the CXCR2 pathway (5, 6, 8). To determine whether CXCL5 directly regulates CXCL13 expression on macrophages through the CXCL5-CXCR2 pathway, we isolated AMs from normal mouse BALF and enriched CD64+ AMs *via* magnetic bead separation. We first confirmed CXCR2 expression on mixed mouse AMs and CD64+ AMs (**Figure 6M**) and then stimulated the CD64+ AMs with H1N1 *in vitro* in the presence or absence of CXCL1, CXCL2, CXCL5, and CXCR2 antagonists. As shown in **Figures 6N, O**, influenza challenge induced considerable expression of CXCL13 mRNA and production of CXCL13 protein in the culture supernatants of the AMs. CXCL1 and CXCL2 expression was also induced in influenza-infected AMs; however, CXCL5 expression was not induced based on the detection of both mRNA and protein levels (**Figures 6N, O** **)**. Furthermore, the levels of CXCL13 in the culture supernatants of influenza-infected macrophages were significantly decreased upon the addition of recombinant CXCL5, recombinant CXCL1 and CXCL2, or all the proteins combined (**Figure 6P**). In contrast, the expression of CXCL13 increased markedly in the group treated with CXCL5 and CXCR2 antagonists compared to the group without the CXCR2 antagonist upon influenza challenge (**Figure 6P**). Together, the *in vitro* data demonstrate that CXCR2 signaling, which is triggered by CXCL5, CXCL1, or CXCL2, is able to suppress CXCL13 expression by CD64+ macrophages upon influenza virus H1N1 infection. Engaged CXCR2 activates several G protein-mediated intracellular signaling pathways, such as the PI3K/AKT and MEK/ERK cascades. To determine the intracellular signaling pathway responsible for the CXCL5-CXCR2 pathway regulating CXCL13 expression, CD64+ AMs were treated with the PI3K inhibitor LY294002 and MEK inhibitor PD98059, respectively, in the presence of CXCL5 upon H1N1 infection. The results showed that inhibition of the PI3K signaling pathway rather than the MEK signaling pathway significantly relieved the inhibitory effect of the cxcl5-cxcr2 axis on CXCL13 expression (**Figure 6P**), indicating that CXCL5-CXCR2 signaling regulates the expression of CXCL13 from AMs through the intracellular PI3K/AKT cascade.

**Figure 7:**
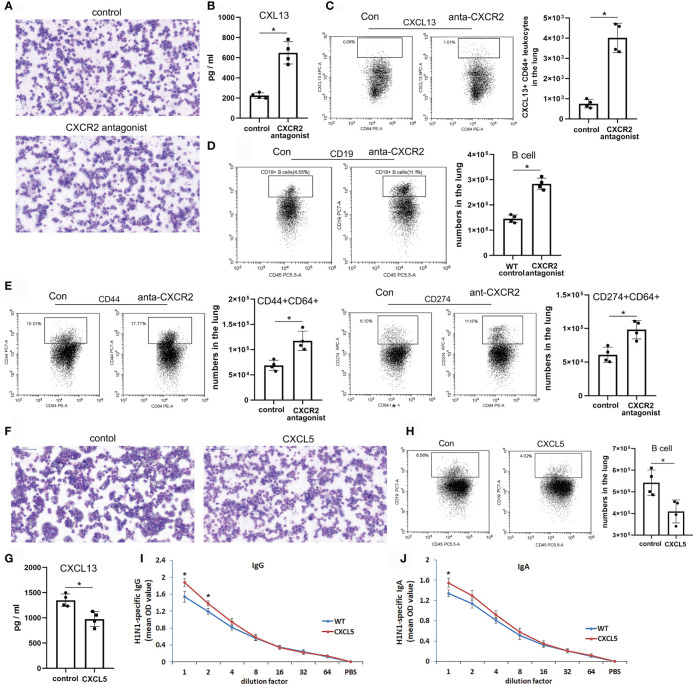
CXCL5-CXCR2 signaling regulated pulmonary neutrophil and B cell recruitment and CXCL13 production from lung macrophages upon H1N1 infection. **(A–E)** WT mice were infected with H1N1 virus (1000 CCID_50_) and treated with either CXCR2 antagonist or PBS as a control during 1-3 d.p.i. Microscopic view of BALF leukocytes from the infected mice collected at 3 d.p.i. and cytospun (×400 magnification) **(A)**. The protein level of CXCL13 in the BALF of the infected mice was measured at 3 d.p.i. by ELISA (n=4) **(B)**. Percentages and numbers of CXCL13-expressing cells among pulmonary CD64+ cells **(C)**, CD19+ B cells **(D)**, and CD44+/CD274+ CD64+ cells **(E)** from the infected mice at 3 d.p.i. by flow cytometry and counting (n=4). **(F–J)** WT mice were infected with H1N1 virus (1000 CCID_50_) and orotracheally instilled with either CXCL5 recombinant protein or PBS as a control during 5-8 d.p.i. Microscopic view of BALF leukocytes from the infected mice collected at 8 d.p.i. and cytospun (×400 magnification) **(F)**. The protein level of CXCL13 in the BALF of the infected mice was measured at 8 d.p.i. by ELISA (n=4) **(G)**. Percentages and numbers of CD19+ B cells from the infected mice at 8 d.p.i. by flow cytometry and counting (n=4) **(H)**. Measurements of H1N1-specific IgG **(I)** and IgA **(J)** in the BALF of the CXCL5 treatment and control mice at 8 d.p.i. (n = 4). The error bars represent the SDs of 4 samples. ^*^P < 0.05 based on Student’s *t*-test.

The above data suggest that CXCR2 signaling, which regulates innate immune neutrophil recruitment, also controls B lymphocyte accumulation in the infected lung upon pulmonary influenza infection. To further prove this, influenza-infected WT mice were treated with a CXCR2 antagonist 1-3 d.p.i., which contributed to notably reduced neutrophil infiltration at 3 d.p.i. in the infected lung (**Figure 7A**). After blockade of CXCR2 signaling, pulmonary expression of CXCL13 was significantly increased in lung CD64+ macrophages/monocytes which highly expressed CD44 and CD274 surface markers at 3 d.p.i. (**Figures 7B, C, E**). Additionally, the number of lung CD19+ B cells was markedly increased in the CXCR2-blockade mouse group at 3 d.p.i. (**Figure 7D**). Moreover, influenza-infected WT mice were intratracheally administered recombinant CXCL5 protein 5-8 d.p.i. to enhance pulmonary CXCL5 levels at the late infection stage. Enhanced and prolonged CXCL5-CXCR2 signaling increased neutrophil numbers in the infected lungs at 8 d.p.i. (**Figure 7F**), while lung B cell numbers and H1N1-specific IgG&IgA levels were decreased together with down-regulated expression of CXCL13 (**Figures 7G-J**). Thus, the data indicate the regulatory role of CXCL5-CXCR2 signaling in both neutrophil and B cell response in the infected lung during H1N1 infection.

### CXCL5 Deficiency Enhanced the Pulmonary B Cell Immune Response and Facilitated Induced Bronchus-Associated Lymphoid Tissue (iBALT) Formation in Infected Lungs

**Figure 8:**
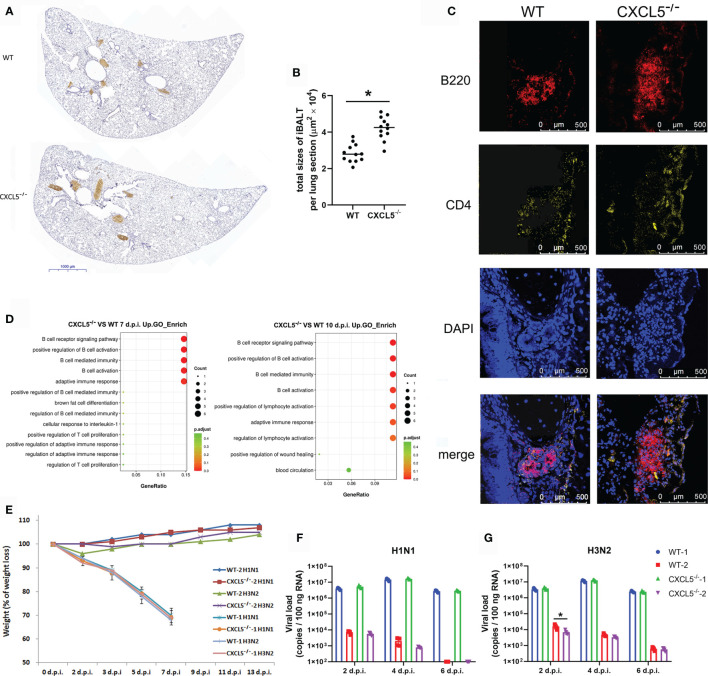
Establishment of adaptive immunity in the infected lungs of WT and CXCL5^-/-^ mice. **(A)** Lung lobe sections from WT and CXCL5^-/-^ mice were stained at 10 d.p.i. with an anti-B220 antibody and hematoxylin (×30 magnification). **(B)** The positively stained area (iBALT structure) in each lung lobe section was calculated using CaseViewer software (3DHISTECH). The total area of iBALT per lung section was calculated from 4 mice per group (3 lung lobe sections per mouse). **(C)** Lung sections were stained with anti-B220 (red) and anti-CD4 (yellow) antibodies for immunofluorescence detection. The immunofluorescence sections were counterstained with DAPI (blue). **(D)** GO functional analysis of upregulated genes in the infected lungs of CXCL5^-/-^ mice compared to WT mice at 7 and 10 d.p.i. The methods and criteria for filtration of differentially expressed genes are described in the MATERIALS AND METHODS section. **(E)** Mice were infected with H1N1 or H3N2 (3000 CCID_50_), and the body weight loss (n=12) of the mice was monitored until 2 weeks post infection. WT-1 H1N1: WT mice infected with H1N1; WT-1 H3N2: WT mice infected with H3N2; CXCL5^-/–^1 H1N1: CXCL5^-/-^ mice infected with H1N1; CXCL5^-/–^1 H3N2: CXCL5^-/-^ mice infected with H3N2; WT-2 H1N1: WT mice recovered from H1N1 infection and infected with H1N1; WT-2 H3N2: WT mice recovered from H1N1 infection and infected with H3N2; CXCL5^-/–^2 H1N1: CXCL5^-/-^ mice recovered from H1N1 infection and infected with H1N1; CXCL5^-/–^2 H3N2: CXCL5^-/-^ mice recovered from H1N1 infection and infected with H3N2. **(F, G)** Lung viral loads of H1N1 **(F)** and H3N2 **(G)**-infected mice were determined based on the number of influenza M gene RNA copies detected by RT-qPCR at different d.p.i. The error bars represent the SDs of 4 samples. ^*^P < 0.05 based on Student’s *t*-test.

The elevated pulmonary B cell and CD138+ plasmablast accumulation and antiviral antibody levels suggested that the virus-specific B cell immune response was enhanced in the infected lungs of CXCL5^-/-^ mice. Indeed, transcriptome analysis of infected lungs at 7 d.p.i. indicated that 60 genes were markedly upregulated in CXCL5^-/-^ mice compared with WT mice (**Supplemental Table 2**). Gene Ontology (GO) and pathway analysis of the upregulated genes showed that they were enriched in 15 biological processes and pathways (**Figure 8D**). Among them, the most activated processes and pathways were related to B cell activation, B cell immunity, and the B cell receptor signaling pathway (**Figure 8D**). The data further demonstrated that B cell activation and the immune response were enhanced upon increased B cell recruitment in the infected lungs of CXCL5^-/-^ mice during the adaptive immunity stage. Immunohistochemistry with a B220 antibody in the infected lungs of both WT and CXCL5^-/-^ mice revealed that B cells accumulated along major bronchial airways and adjacent to perivascular spaces at 10 d.p.i. (**Figure 8A**). Further immunofluorescence detection showed that B cells accumulated in follicle structures surrounding CD4 T cells near the airways in the lungs (**Figure 8C**). B cell formation in the lungs during the late infection stage likely indicates the development of mucosal lymphoid tissue called iBALT, a type of ectopic lymphoid tissue that is formed upon inflammation or infection, such as influenza virus infection, in both mice and humans (30). iBALT is considered a tertiary lymphoid structure whose formation is induced in peripheral tissues such as lung tissue under conditions of severe inflammatory diseases (30). The formation of pulmonary iBALT during the late infection recovery stage is referred to as effective local adaptive immunity against related pathogens and maintains memory cells for successive reinfections (30, 31). As shown in **Figures 8A, B**, the total area of iBALT in the infected lungs was significantly greater in CXCL5^-/-^ mice than in WT mice, which suggests advanced formation of iBALT in CXCL5^-/-^ mice. Transcriptome analysis of infected lungs at 10 d.p.i. revealed that the B cell immune response and adaptive immunity were more vigorous in CXCL5^-/-^ mice than in WT mice (**Figure 8D** and **Supplemental Table 2**). Together, the above data indicate that the local B cell immune response and adaptive immunity were enhanced in the infected lungs of CXCL5^-/-^ mice during the late infection and recovery stages. Considering that the different extents of iBALT formation may affect pulmonary antiviral immunity after reinfection with influenza virus, the recovered WT and CXCL5^-/-^ mice were reinfected using the same influenza virus (H1N1) or a different influenza virus (H3N2). The results showed that a lethal dose (3000 CCID_50_) of either H1N1 or H3N2 resulted in the death of all mice that had not been exposed to influenza virus within 7 d (**Figure 8E**). In contrast, all WT and CXCL5^-/-^ mice that had recovered from H1N1 infection (40 d after 14 d.p.i.) and were infected with the lethal dose of either H1N1 or H3N2 survived beyond 14 d.p.i. No obvious weight loss was observed, although minor weight loss was observed in the WT mice infected with H3N2 (2 d.p.i., 5% weight loss) (**Figure 8E**). Viral load detection showed that both the WT and CXCL5^-/-^ reinfected mice with either H1N1 or H3N2 challenge remarkably restrained viral infection and replication (**Figures 8F, G** **)**. There was no significant difference in virus replication level between WT and CXCL5^-/-^ reinfected mice, except that the virus load of the CXCL5^-/-^ mice was lower than that of the WT mice at 2 d.p.i. after H3N2 infection (**Figure 8G**). Together, the results demonstrated that after recovery, both H1N1-infected WT and CXCL5^-/-^ mice acquired potent adaptive immunity against infection with the same influenza strain or a different influenza strain (H3N2).

## Discussion

CXCL5, a neutrophil chemoattractant, is involved in a variety of human inflammatory diseases and in tumorigenesis (10–12). In terms of respiratory pathogen infection, it plays an unexpected role in chemokine scavenging to regulate pulmonary host defense against *E.coli* infection (14). Recently, in a SARS-CoV-2-infected hACE2 -transfected mouse model, we found it affects neutrophil chemotaxis in infected lungs during the early infection stage, which suggests that CXCL5 functions in respirovirus infection (32). In the influenza virus infection mouse model described here, H1N1 infection induced CXCL5 production in infected lung during the acute infection period (**Figures 1C, E, F** **)**. Upon infection, influenza virus first infects and proliferates in airway epithelial cells and alveolar epithelial cells. CXCL5 is produced by lung tissue cells after infection based on data from chimeric mice, and it may be expressed by the alveolar type II epithelial cells (13). In addition to production by structural cells, large amounts of CXCL5 production by blood hematopoietic cells were observed upon influenza infection (**Figure 1D-F**). CXCL5 is normally present in the blood (at approximately 200 pg/ml) in mice (**Figure 1D**), where it is produced and released by activated platelets (14). A recent breakthrough study found that the lungs are reservoir sites for platelet progenitors and platelet biogenesis (33), and influenza infection is known to induce platelet activation in infected mouse lungs (34). It seems that pulmonary influenza infection activates lung vascular platelets to produce CXCL5, which, together with CXCL5 expressed by lung tissue cells, exerts immunoregulatory functions.

While neutrophil influx into infected lungs was increased in the *E. coli*-infected CXCL5^-/-^ mouse model, the data suggest that it was decreased in the influenza-infected CXCL5^-/-^ mouse model during the early innate immunity stage. In contrast to the findings obtained with the bacterial infection model, those obtained with the influenza-infected CXCL5^-/-^ mice indicated that the levels of the major neutrophil chemoattractants CXCL1 and CXCL2 in the lungs were significantly decreased during the early infection stage (**Figure 6A**). Together with CXCL5 deficiency, the impaired neutrophil recruitment in infected lungs was largely due to low levels of pulmonary neutrophil chemokines. It is possible that expression of CXCL1/2 was decreased from the infected lung of CXCL5-/- infected mice due to the down-regulated CXCR2 axis by CXCL5 deficiency. A work from Tavares LP, et al. revealed that antagonism of CXCR2 axis reduced pulmonary CXCL1/2 expression upon influenza infection (35). Considering abundant expression of CXCL5 in the infected lung during early influenza infection stage, CXCL5 deficiency might down-regulate CXCR2 axis in lung epithelial cells and lead to reduced CXCL1/2 expression form the cells which in turn decreasing neutrophil infiltration. In addition, DARC, as a scavenging receptor for CXCL1, CXCL2 and CXCL5, is present on both erythrocytes and postcapillary venular endothelial cells (9). The lack of CXCL5 may have led to increased CXCL1 and CXCL2 scavenging by pulmonary capillary DARC and contributed to the downregulation of the two neutrophil chemokines in infected lungs. Also, it is possibly due to reduced neutrophil numbers after infection in the infected lungs considering that neutrophils are also major producers of CXCL1 and CXCL2 (4, 5).

In addition to regulating neutrophil chemokine levels in the influenza infection mouse model, CXCL5 has also been found to regulate the expression of another chemokine, CXCL13 (**Figure 6C**), a predominant B cell chemoattractant, in infected lungs after influenza infection (20, 21, 26). CXCL13 has been reported to be expressed in infected lungs after influenza infection, and lung fibroblasts are the primary cells of CXCL13 production 5 d.p.i (20).. We further found that CXCL13 was expressed mainly by lung tissue cells during the early infection stage (3 d.p.i.); however a large proportion of CXCL13 was expressed by lung leukocytes during the late infection stage (8 d.p.i.). In the case of CXCL5^-/-^ infected mice, the expression of CXCL13 increased beginning at 3 d.p.i.; the increase was mostly attributable to pulmonary leukocytes instead of tissue cells (**Figure 6E**). Pulmonary CD64+ macrophages/monocytes expressed CXCL13 from 3 d.p.i. to 8 d.p.i. in CXCL5^-/-^ infected mice, while in WT infected mice, CXCL13 was not expressed at 3 d.p.i. (**Figures 6G, H**). The different expression patterns of CXCL13 between WT and CXCL5^-/-^ infected mice indicated that CXCL5 affected CXCL13 expression in pulmonary macrophages/monocytes upon influenza virus infection. Further *in vitro* studies demonstrated that influenza virus infection induced CXCL13 expression in AMs and that CXCL5 administration inhibited CXCL13 expression in influenza-infected AMs (**Figure 6P**). The findings that administration of CXCL1 and CXCL2 also suppressed CXCL13 expression in influenza-infected AM and that blockade of CXCR2 binding relieved the inhibitory effect of CXCL5 on the expression of CXCL13 in infected AMs (**Figure 6P**) suggest that activation of the CXCL1/2/5-CXCR2 pathway can impair influenza infection-induced CXCL13 expression in pulmonary macrophages. Recently, work by Bellamri et al. revealed that TNF-α and IL-10 optimize CXCL13 expression in AMs (24); thus, it is possible that the activated CXCR2 pathway may inhibit CXCL13 expression by controlling TNF-α/IL-10 production by influenza-infected AMs, in turn limiting the positive regulation of TNF-α/IL-10 on CXCL13 production in AMs. In our study, there were no significant differences in the protein levels of TNF-α and IL-10 in the culture supernatants of influenza-infected AMs in the presence or absence of the CXCL5 protein (data not shown). Therefore, it is likely that activated CXCR2 signaling restricts CXCL13 expression in influenza-infected AMs *via* the intracellular pathway, especially the PI3K/AKT cascade.

The discovery that CXCL13 expression is controlled by CXCL5 signaling in the context of pulmonary influenza infection was unexpected. Specifically, the signal from the CXCL1/2/5-CXCR2 axis regulated the expression of CXCL13 in macrophages. CXCL1, CXCL2, and CXCL5 are predominant neutrophil chemoattractants that recruit neutrophils and mediate inflammation *via* the receptor CXCR2. The functions of these IL-8 family chemokines in the immune response to pathogens mainly involve the control of bacterial infection and the antiviral innate immune response (4, 6). CXCL13 has been reported to play a vital role in the recruitment of B cells and the formation of the ectopic germinal center (GC) (also called iBALT) in infected lungs during adaptive immunity and recovery upon severe pulmonary influenza infection (20, 36). It seems that the CXCL1/2/5-CXCR2 axis plays regulatory roles in controlling pulmonary innate-to-adaptive immune progression. In the influenza H1N1 infection mouse model, we revealed that the neutrophil chemokines CXCL1, CXCL2, and CXCL5 were expressed mainly during the innate immunity stage, while an increase in the expression of the B lymphocyte chemokine CXCL13 followed a decrease in the expression of CXCL1/2/5 and dominated in the late adaptive immunity stage (**Figures 6A, C** **)**. Downregulation of the CXCL1/2/5-CXCR2 axis, as demonstrated in the CXCL5^-/-^ infection mouse model and the CXCR2 blockade model, enhanced CXCL13 expression in infected lungs as soon as during the innate immunity stage (**Figures 6A, C** and **Figures 7B, C** **)**. It is likely that during the innate immunity stage of fatal influenza H1N1 infection, infected lung epithelial cells initially produce chemokines related to innate immunity, such as the neutrophil chemokines CXCL1, CXCL2, and CXCL5, to recruit neutrophils into the infected lungs and exert an antiviral innate immune response. In addition, CXCL1, CXCL2, and CXCL5 suppress excess CXCL13 expression in macrophages *via* the CXCR2 axis during the early infection stage. As the infection progresses, the expression of CXCL1/2/5 decreases and in turn upregulates the expression of CXCL13 by macrophages, monocytes, and DCs, which contributes to B lymphocyte accumulation in the infected lungs during the late infection and recovery stages. The findings suggest that macrophages are the key leukocytes in the pathway by which CXCL5 controls CXCL13 expression during influenza infection. During influenza infection in the current study, macrophages were abundant in the infected lungs during the innate immunity stage, but their abundance was decreased in the adaptive immunity stage, causing macrophages to account for relatively small proportion of total pulmonary leukocytes (**Figures 4B, C** **)**. CXCL13 is reported to be expressed by macrophages upon lipopolysaccharide (LPS) stimulation (24), and M2-phenotype macrophages have also been described as cellular sources of CXCL13 (28, 29). In the current study, the phenotype of CD64+ macrophages from CXCL5^-/-^ infected mice was different from that of CD64+ macrophages from WT infected mice at the same infection time point (3 d.p.i.). Three surface markers, CD44, CD206, and CD274, were highly expressed on CD64+ macrophages of CXCL5^-/-^ infected mice, and CD44 and CD274 expression was significantly upregulated in these macrophages compared with the macrophages from WT infected mice (**Figures 6I–K**). CD44 has been reported to resolve lung inflammation and to act as a negative regulator of LPS-TLR signaling in mouse macrophages (37, 38). CD274 is considered a critical regulator of M2 macrophage polarization (39). The observed high expression of these surface markers indicated that a reduced CXCR2 signaling caused pulmonary macrophages surrounded by an innate immune microenvironment induced by influenza infection to convert into M2-like macrophages that possibly produced CXCL13. The CXCR2 blockade infection model further supported this notion (**Figure 7**). It seems that the pulmonary immune system controls abundant CXCL13 expression from a large number of macrophages during the anti-influenza innate immune response.

Increased B cell recruitment to the infected lungs led to an enhanced local pulmonary B cell immune response in CXCL5^-/-^ infected mice. Typically, B cells are activated in the MLNs, where B cells under antigen priming, somatic hypermutation, and affinity-based selection within GCs; mature B cells or plasma cells are then released into the circulation, where they produce antibodies (3). However, after pulmonary influenza virus infection, the lungs begin to support B cell and T cell responses, resulting in the accumulation of T and B lymphocytes and the formation of ectopic GCs (iBALT) to provide protective humoral responses at these sites (31). In the current study, the B cell immune response derived from the MLN activation pathway was not improved in CXCL5^-/-^ mice compared to WT mice (**Figures 5I, M** **)**. The enhancement of the local pulmonary B cell immune response was most likely due to a GC-independent pathway. B cells in the infected lungs underwent substantial B cell activation and maturation, as demonstrated by transcriptome analysis of the infected lung tissues (**Figure 8D** and **Supplementary Excel**). Thus, the enhanced pulmonary B cell immune response was attributable to infiltrated lung B cells, the numbers of which were increased in CXCL5^-/-^ infected mice. In addition, increased B cell recruitment and accumulation in the infected lungs of CXCL5^-/-^ mice also contributed to increased iBALT formation during the late infection and recovery stages (**Figures 8A, B** **)**. Once formed, iBALT probably plays an important role in combating successive rounds of the same type of infection and may help initiate local immunity to different subtypes of viruses or even to unrelated pathogens (30). The increased formation of iBALT in CXCL5^-/-^ infected mice in the present study did not reduce disease development upon reinfection with H1N1, even upon reinfection with a fatal dose of a different subtype of influenza virus, H3N2 (**Figures 8E–G**). The WT infected mice likely acquired adequate immunity against the second rounds of infection by the different viruses. Under challenge with the lethal doses that we used, it seemed that normal mice were able to exhibit potent immune responses with iBALT formation to deal with reinfection by influenza virus even a different subtype of influenza virus. On this basis, we conclude that increased iBALT formation is not necessary for adaptive immunity against the same virus. On the other hand, the enhanced formation of iBALT after influenza infection may also cause adverse effects considering that iBALT plays pathological roles in chronic pulmonary diseases (40). Collectively, these results suggest that although a lack of CXCL5 alleviates lung inflammation during the early stage and affects viral clearance to some extent during the late stage of influenza virus infection in mice, it does not obviously improve the overall outcome of viral infection in terms of survival rate, viral clearance time, lung inflammation development or establishment of adaptive immunity. The role of CXCL5 in pulmonary influenza infection is not redundant; rather, CXCL5 helps orchestrate antiviral innate and adaptive immunity.

## Data Availability Statement

The raw data supporting the conclusions of this article will be made available by the authors, without undue reservation.

## Ethics Statement

Animal studies were reviewed and approved by the animal ethics committee of the Institute of Medical Biology, CAMS.

## Author Contributions

Conceptualization: LG and LL. Investigation: LG, NL, ZY, JY, YC, HL, HZ, and XZ. Data Analysis: LG, NL, and LL. Writing-original draft: LG. Writing-review and editing: LL, NL, and HS. Material/Reagents: JM and GW. Funding acquisition: JM, LL, and LG. All authors contributed to the article and approved the submitted version.

## Funding

This work was supported by grants from the National Natural Science Foundation of China (31570900, 82041017, 81560262, and 81960294), the Yunnan Key Laboratory of Children’s Major Disease Research (202005AG070073), and the Kunming Health Science and Technology Talent Project-10 Projects (2020-SW(P)-11).

## Conflict of Interest

The authors declare that the research was conducted in the absence of any commercial or financial relationships that could be construed as a potential conflict of interest.

## Publisher’s Note

All claims expressed in this article are solely those of the authors and do not necessarily represent those of their affiliated organizations, or those of the publisher, the editors and the reviewers. Any product that may be evaluated in this article, or claim that may be made by its manufacturer, is not guaranteed or endorsed by the publisher.
