# Supplementary material for: Role of CXCL5 in Regulating Chemotaxis of Innate and Adaptive Leukocytes in Infected Lungs Upon Pulmonary Influenza Infection
Source: Front Immunol. 2021 Nov 18;12:785457. doi: 10.3389/fimmu.2021.785457 (PMC8637413; doi:10.3389/fimmu.2021.785457)
Supplement: Supplementary file 1 [file DataSheet_1.pdf]

Supplemental Figure 1. Gating strategy used for CyTOF analysis (A) and flow cytometry (B).

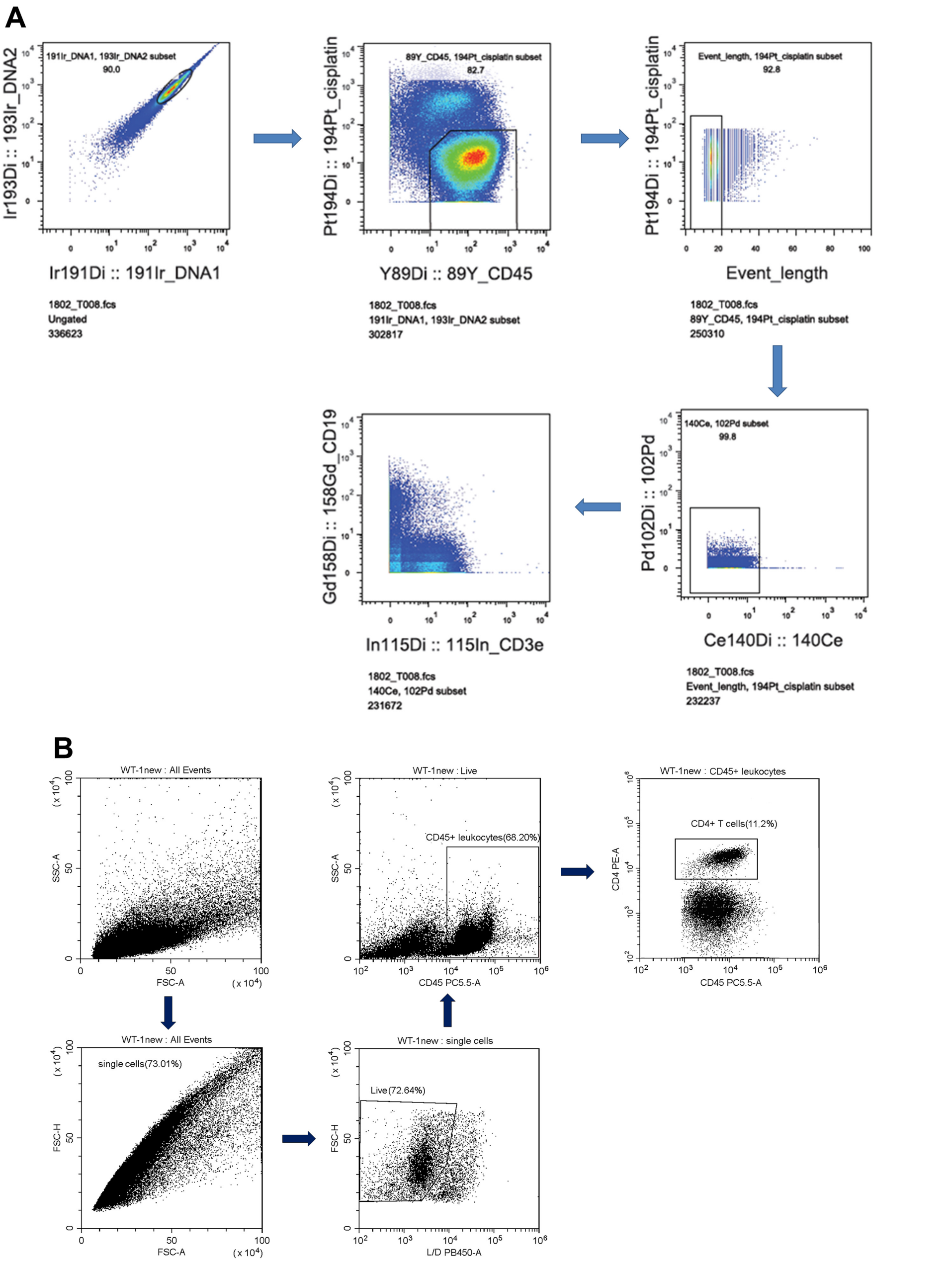

Supplemental Figure 2. Illustration of the expression of 42 cellular markers in 38 cell clusters in normal mouse lungs as determined by CyTOF analysis. Clusters corresponding to different leukocyte subsets are framed in red. The heatmap indicates the expression levels of the different markers.

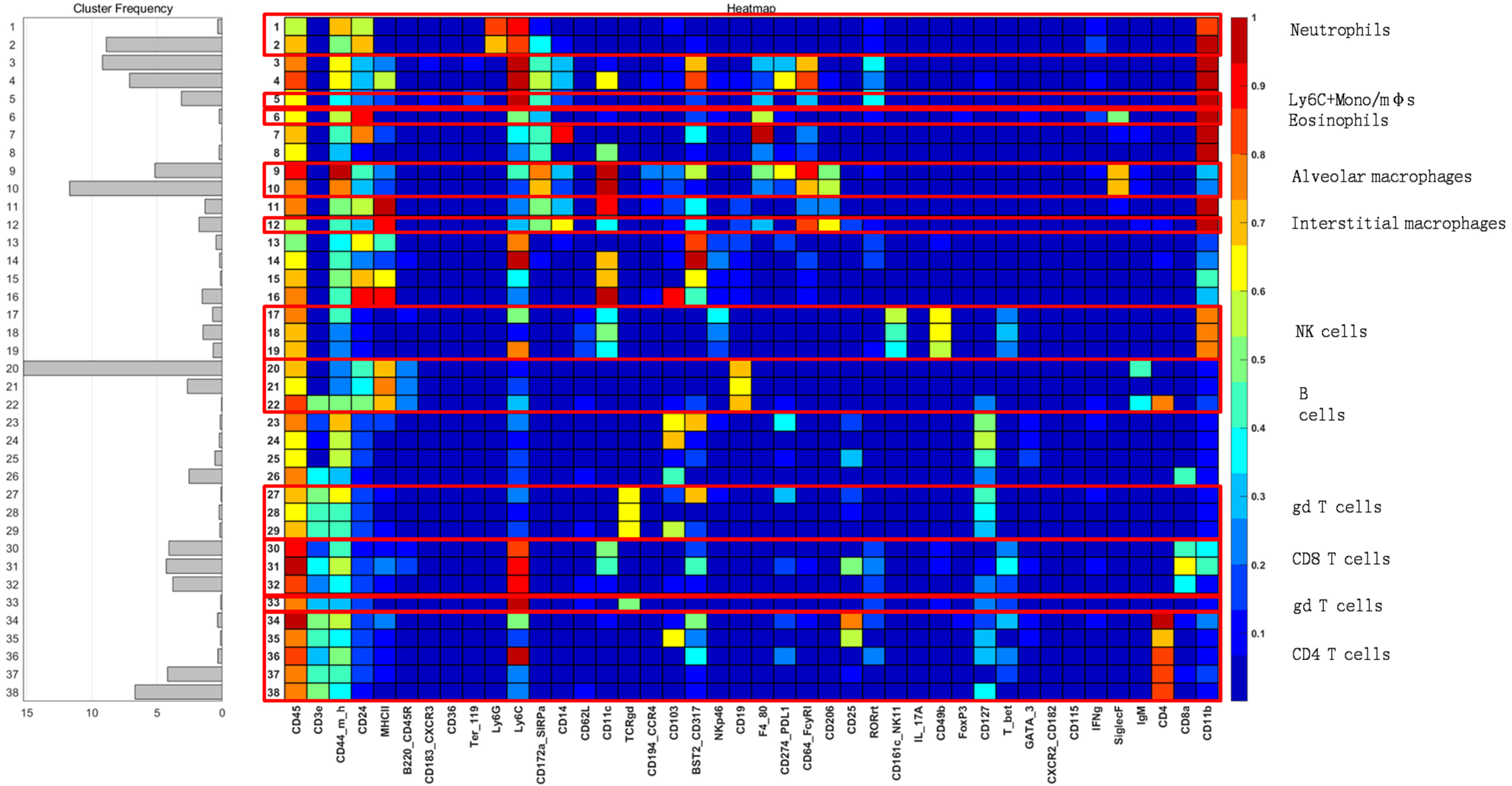

Supplemental Figure 3. H1N1-specific antibody levels detection. (A) HI titers of the sera from WT and CXCL5-/- mice at 8 d.p.i. against H1N1 virus were evaluated (n=4). (B) Measurements of influenza-specific IgA in the BALF of WT and CXCL5-/- mice infected with the H1N1 virus at 8 d.p.i. (n = 4). The error bars represent the SDs. *P<0.05 based on Student's t-test.

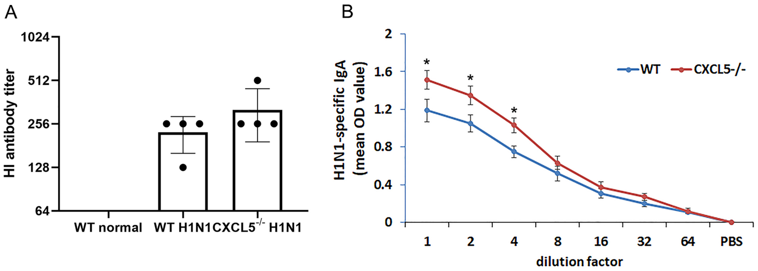

No.	Label	Antibody	Clone	Vender	Target
**1**	89Y	CD45	30-F11	BioLegend	surface
**2**	115ln	CD3e	145-2C11	BioLegend	surface
**3**	139La	CD44	IM7	BioLegend	surface
**4**	141Pr	CD24	M1/69	BioLegend	surface
**5**	142Nd	MHC II	Y-3P	BioXcell	surface
**6**	143Nd	CD45R[B220]	RA3-6B2	BioLegend	surface
**7**	144Nd	CD183[CXCR3]	CXCR3-173	BioLegend	surface
**8**	145Nd	CD36	HM36	BioLegend	surface
**9**	146Nd	TER-119	TER-119	BioLegend	surface
**10**	147Sm	Ly-6G	1A8	BioLegend	surface
**11**	148Nd	Ly-6C	HK1.4	BioLegend	surface
**12**	149Sm	CD172a[SIRPα]	P84	BioLegend	surface
**13**	150Nd	CD14	Sa14-2	BioLegend	surface
**14**	151Eu	CD62L	MEL-14	BioLegend	surface
**15**	152Sm	CD11c	N418	BioLegend	surface
**16**	153Eu	TCRgd	GL3	BioLegend	surface
**17**	154Sm	CD194[CCR4]	2G12	BioLegend	surface
**18**	155Gd	CD103	2.00E+07	BioLegend	surface
**19**	156Gd	CD317[BST2]	44E9R	BioLegend	surface
**20**	157Gd	CD335[NKp46]	29A1.4	BioLegend	surface
**21**	158Gd	CD19	6D5	BioLegend	surface
**22**	159Tb	F4/80	Cl:A3-1	Bio-Rad	surface
**23**	160Gd	CD274[PD-L1]	10F.9G2	BioLegend	surface
**24**	161Dy	CD64[FcγRI]	X54-5/7.1	BioLegend	surface
**25**	162Dy	CD206[MMR]	C068C2	BioLegend	intra
**26**	163Dy	CD25[IL-2R]	3C7	BioLegend	surface
**27**	164Dy	RORgt	600214	R&D	intra
**28**	165Ho	CD161c[NK1.1]	PK136	BioLegend	surface
**29**	166Er	IL-17A	TC11-18H101	BioLegend	intra
**30**	167Er	CD49b	DX5	BioLegend	surface
**31**	168Er	FoxP3	FJK-16s	eBioscience	intra
**32**	169Tm	CD127[IL-7Rα]	A7R34	BioLegend	surface
**33**	170Er	T-bet	4B10	BioLegend	intra
**34**	171Yb	GATA3	TWAJ	eBioscience	intra
**35**	172Yb	CD182[CXCR2]	SA044G4	BioLegend	surface
**36**	173Yb	CD115[CSF-1R]	AFS98	BioLegend	surface
**37**	174Yb	IFNg	XMG1.2	BioXcell	intra
**38**	175Lu	CD170[Siglec-F]	E50-2440	BD	surface
**39**	176Yb	IgM	RMM-1	BioLegend	surface
**40**	197Au	CD4	RM4-5	BioLegend	surface
**41**	198Pt	CD8a	53-6.7	BioLegend	surface
**42**	209Bi	CD11b	M1/70	BioLegend	surface

Supplementary Table 1. The 42 antibodies used in CyTOF analysis.
